# Serine/threonine kinase 33 mediates thrombin-induced interleukin-8 release from human lung epithelial cells in severe asthma

**DOI:** 10.1186/s12931-025-03368-6

**Published:** 2025-10-21

**Authors:** Wun-Hao Cheng, Wen-Shan Chang, Po-Hao Feng, Kuan-Yuan Chen, Kang-Yun Lee, Yu-Chih Wu, Lee-Yuan Lin, Fara Silvia Yuliani, Chien-Huang Lin, Bing-Chang Chen

**Affiliations:** 1 School of Respiratory Therapy, College of Medicine, Taipei Medical University, 250 Wu-Hsing Street, Taipei, 11031 Taiwan; 2 Respiratory Therapy, Division of Pulmonary Medicine, Wan Fang Hospital, Taipei Medical University, Taipei, Taiwan; 3 Chen Wei-Tien Research Center for Thoracic Medicine, College of Medicine, Taipei Medical University, Taipei, Taiwan; 4 Master program in Thoracic Medicine, School of Respiratory Therapy, Taipei Medical University, Taipei, Taiwan; 5 Division of Pulmonary Medicine, Shuang Ho Hospital, Taipei Medical University, Taipei, Taiwan; 6 Department of Pharmacology and Therapy, Faculty of Medicine, Public Health, and Nursing, Universitas Gadjah Mada, Yogyakarta, Indonesia; 7 School of Medicine, College of Medicine, Taipei Medical University, Taipei, Taiwan; 8 Graduate Institute of Medical Sciences, College of Medicine, Taipei Medical University, 250 Wu-Hsing Street, Taipei, 11031 Taiwan

**Keywords:** Severe asthma, STK33, Thrombin, IL-8/CXCL8, C-Myc, Epithelial cell

## Abstract

Interleukin (IL)-8, also known as chemokine (CXC motif) ligand 8, contributes to the pathogenesis of neutrophilic asthma. Thrombin exacerbates IL-8/CXCL8-mediated inflammatory responses. However, the underlying mechanisms remain unclear. Phosphorylation of serine/threonine kinase 33 (STK33) by extracellular signal-regulated kinase (ERK) has been implicated in lung cancer metastasis, but its role in thrombin-induced airway inflammation remains to be determined. In this study, we investigated whether STK33 mediates thrombin-induced IL-8/CXCL8 release from airway epithelial cells in severe asthma. Single-cell RNA sequencing revealed upregulated STK33 expression in bronchial epithelial cells from patients with severe asthma. Immunofluorescence staining indicated increased coexpression of STK33 and macrophage inflammatory protein 2 (murine homolog of IL-8/CXCL8) in the airway epithelial cells of mice with ovalbumin- and house dust mite–induced asthma. Thrombin induced STK33 phosphorylation at serine residues. Notably, STK33 knockdown markedly inhibited thrombin-induced IL-8/CXCL8 release from A549 and BEAS-2B cells. Thrombin also induced the formation of an ERK–STK33 complex, and STK33 knockdown reduced thrombin-induced ERK phosphorylation. Colocalization of STK33, p-c-Myc, and thrombin was increased in asthmatic mice. Moreover, thrombin increased the phosphorylation of c-Myc in A549 cells; this increase in c-Myc phosphorylation was significantly attenuated by ERK inhibition (U0126 treatment) and STK33 knockdown. Furthermore, thrombin promoted the binding of c-Myc to the IL-8/CXCL8 promoter, and c-Myc knockdown suppressed thrombin-induced IL-8/CXCL8 release from A549 and BEAS-2B cells. Collectively, these findings indicate that thrombin induces IL-8/CXCL8 release through the ERK/STK33/c-Myc pathway, highlighting STK33 as a potential therapeutic target in severe asthma.

**Supplementary Information:**

The online version contains supplementary material available at 10.1186/s12931-025-03368-6.

## Introduction

Asthma is a chronic inflammatory lung disease characterized by variable expiratory airflow limitation and clinical manifestations such as airway hyperresponsiveness, mucus hyperproduction, dyspnea, wheezing, chest tightness, and persistent airway inflammation [1]. Central to asthma pathophysiology is chronic airway inflammation. Airway epithelial cells serve as both structural and immunological barriers against environmental insults. Exposure to environmental stimuli such as particulate matter and allergens can damage these cells, enhancing the release of epithelial-derived proinflammatory cytokines—for example, interleukin (IL)−8, also known as chemokine (CXC motif) ligand 8—and initiating downstream inflammatory cascades that drive asthma progression [2–4].

IL-8/CXCL8 is a key chemokine that facilitates neutrophil recruitment to sites of inflammation, contributing to the pathogenesis of neutrophilic asthma—a subtype for which no effective therapeutic strategies are yet available [2, 5]. Notably, elevated IL-8/CXCL8 expression was observed in airway secretions from patients with severe asthma and is associated with increased neutrophil infiltration into airway epithelial cells [6]. Therefore, elucidating the mechanisms underlying IL-8/CXCL8 expression during airway inflammation in asthma is crucial for identifying potential therapeutic targets.

Thrombin is a serine protease involved in the coagulation cascade [7]. In addition to facilitating the conversion of fibrinogen to fibrin, thrombin exacerbates allergic inflammation and airway hyperresponsiveness [8, 9]. Epithelial damage leads to the release of thrombin, which then binds to protease-activated receptors on lung epithelial cells. Activation of these receptors induces the secretion of various inflammatory mediators, such as IL-8/CXCL-8, IL-6, and prostaglandin E_2_, resulting in chronic airway inflammation [10, 11]. Studies have revealed elevated thrombin levels in the airways of patients with asthma [12, 13]. Airway stimulation with thrombin markedly increases the level of macrophage inflammatory protein 2 (MIP-2), the murine homolog of human IL-8/CXCL8, in bronchoalveolar lavage fluid [10]. These findings underscore the pivotal role of thrombin in driving airway inflammatory responses in asthma.

Serine/threonine kinase 33 (STK33) is a serine kinase predominantly expressed in testicular epithelial cells, lung epithelial cells, and alveolar macrophages [14, 15]. Phosphorylation of STK33 at serine 496 regulates cell cycle progression in HeLa cells [16, 17]. Phosphorylation of STK33 by extracellular signal-regulated kinase (ERK) leads to dynamic cytoskeletal rearrangements, which facilitates metastasis, particularly in lung and colorectal cancers [15]. Our previous analysis of single-cell RNA sequencing (scRNA-seq) data from patients with asthma revealed significantly upregulated *STK33* expression in bronchial epithelial cells. However, the role of STK33 in thrombin-induced IL-8/CXCL8 expression and its relevance to asthma pathogenesis remain unexplored.

Notably, c-Myc is a pleiotropic transcription factor that regulates cell proliferation, cell differentiation, cell cycle progression, and apoptosis, facilitating the pathogenesis of various cancers [18–20]. Hypoxia enhances the activity of c-Myc through hypoxia-inducible factor 2α, thereby upregulating IL-8/CXCL8 expression in human endothelial cells [21]. In patients with asthma, c-Myc expression is considerably upregulated in type 2 innate lymphoid cells. Allergen exposure triggers airway epithelial cells to release cytokines such as IL-25 and IL-33, which subsequently induce c-Myc expression, thereby promoting airway inflammation, mucus hyperproduction, and airway hyperresponsiveness [22]. Collectively, these findings implicate c-Myc in the expression of IL-8/CXCL8 in epithelial cells and the pathological progression of asthma. STK33 enhances the transcriptional activity of c-Myc, thereby regulating the proliferation and differentiation of hepatocellular carcinoma cells [23]. However, whether thrombin induces IL-8/CXCL8 expression through STK33-mediated activation of c-Myc remains unclear.

In this study, we investigated whether STK33 mediates thrombin-induced IL-8/CXCL8 release from lung epithelial cells in severe asthma. The results revealed that STK33 expression was upregulated in airway epithelial cells from patients with severe asthma. Furthermore, thrombin induced the formation of an ERK–STK33 complex, leading to the phosphorylation and activation of c-Myc, which in turn increased the expression and release of IL-8/CXCL8 from human lung epithelial cells.

##  Materials and methods

### Materials

The human lung epithelial cell line A549 (CCL-185) and the human bronchial epithelial cell line BEAS-2B (CRL-9609) were obtained from the American Type Culture Collection (Manassas, VA, USA). Antibiotic–antimycotic solution (30-004-CI) was purchased from Corning Mediatech (Manassas, VA, USA). ERK inhibitor (U0126), α-tubulin antibody (T9026), thrombin (T4648), aluminum hydroxide, ovalbumin, control small interfering RNA (scrambled siRNA), STK33 siRNA, and c-Myc siRNA were obtained from Sigma-Aldrich (St. Louis, MO, USA). NovaHisto (20X; LB0200-0100) was acquired from Bionovas (Toronto, ON, Canada). IL-8/CXCL8 antibody (bs-0780R), and thrombin activation peptide fragment 1 antibody, Cy5 conjugated (bs-10387R-Cy5) were purchased from Bioss (Woburn, MA, USA). Anti-STK33 antibody (ab237759), Goat Anti-Rabbit Immunoglobulin (Ig)G H&L Alexa Fluor 555 (ab150078), Goat Anti-Rabbit IgG H&L Alexa Fluor 488 (ab150077) secondary antibodies, and mounting medium with 4′,6-diamidino-2-phenylindole-aqueous, fluoroshield (ab104139) were obtained from Abcam (Cambridge, UK). Antibodies against ERK1/2 (sc-514302), phosphorylated ERK (sc-7383), and STK33 (sc-376498) as well as horseradish peroxidase–conjugated anti-rabbit IgG, anti-mouse IgG, protein G beads, and anti-mouse IgG (sc-2314) were obtained from Santa Cruz Biotechnology (Dallas, TX, USA). c-Myc antibody (PK-9; IRM309) was purchased from iREAL Biotechnology (Hsinchu City, Taiwan). Phosphorylated c-Myc (Ser62) polyclonal antibody (PA5-104729), Lipofectamine 3000, Dulbecco’s Modified Eagle Medium (DMEM)/Nutrient Mixture F-12 (F-12), and fetal bovine serum were acquired from Invitrogen Life Technologies (Carlsbad, CA, USA). A chromatin immunoprecipitation (ChIP) assay kit was obtained from Upstate Biotechnology Millipore (Lake Placid, NY, USA). Opti-MEM I Reduced Serum Medium (31985070) was purchased from Gibco (Waltham, MA, USA).

### Human lung tissue collection

Population selection criteria have been described previously [24]. Participants included individuals with severe asthma, characterized by persistently reduced lung function (post-bronchodilator FEV₁ < 60% of predicted), as well as healthy controls with preserved pulmonary function (FEV₁ >80% of predicted). Airway tissue samples were collected from patients with severe asthma through bronchoscopy. The samples were subjected to immunohistochemical staining. Informed consent was obtained from all patients.

### Single-Cell RNA sequencing (scRNA-seq) of human airway tissue

Healthy control tissue was obtained from the trachea of a non-smoker, as referenced in the previously published dataset GSE134174 [25]. Severe asthma airway tissue biopsies were collected from five patients and immediately processed for scRNA-seq. The tissue samples were transferred to ice-cold DMEM containing collagenase, proteinase, and DNase I and enzymatically dissociated at 37 °C with gentle agitation to obtain single-cell suspension. The suspension was passed through a 70-µm cell strainer to remove debris and undigested tissue fragments. After red blood cell lysis and washing, cell viability and density were evaluated through trypan blue exclusion. Single-cell libraries were prepared using the Chromium Single Cell 3’ Gene Expression platform (version 3; 10x Genomics), following the manufacturer’s instructions. After complementary DNA synthesis, amplification, and library construction, the samples were sequenced on an Illumina NovaSeq 6000 platform to ensure adequate read depth per cell. Sequencing data were processed using the Cell Ranger pipeline (10x Genomics) for demultiplexing, alignment to the human reference genome (GRCh38), and gene calculation. Downstream analysis was performed using the Seurat package (version 5.0) in R. Quality control criteria were set as nFeature_RNA >200, nFeature_RNA < 2500, and percent.mt < 5 to exclude low-quality cells. After quality control and normalization, principal component analysis and t-distributed stochastic neighbor embedding were performed for dimensionality reduction and visualization. Cell clustering and marker identification were performed using Seurat’s built-in functions.

### Gene annotation of scRNA-seq

Gene annotation was performed regarding a previous study [26]. In this study, we employed scROSHI (single-cell RNA annotation by Optimized Self-training with Highly Informative genes) for annotating the scRNA-seq data. scROSHI is an annotation method that leverages optimized self-training with a curated set of highly informative genes to enhance both the accuracy and efficiency of single-cell data annotation.

### Establishment of a mouse model of ovalbumin-induced asthma

C57BL/6 mice were provided by National Laboratory Animal Center (NARLabs, Taiwan).

On days 1, 8, and 15, C57BL/6 mice were intraperitoneally injected with 10 µg of ovalbumin and 4 mg of aluminum hydroxide (alum) in 0.1 mL phosphate-buffered saline (PBS). The mice were exposed to aerosolized PBS or 5% ovalbumin twice a week for 8 weeks (from week 4 to week 11). At week 12, the mice were euthanized to obtain lung tissue and bronchoalveolar lavage fluid.

### Establishment of a mouse model of house dust mite-induced asthma

BALB/c mice were provided by National Laboratory Animal Center (NARLabs, Taiwan). BALB/c mice were intratracheally administered HDM extract (100 µg on days 0 and 7; 30 µg on days 21–23) and sacrificed on day 24. PBS-treated mice served as controls.

### Cell culture

A549 and BEAS-2B cells were cultured in DMEM/F-12 supplemented with 10% fetal bovine serum and 1% antibiotic–antimycotic solution; the cells were maintained at 37 °C under 5% CO_2_ (Lin et al., 2006). For different experiments, different culture plates were selected with consideration of the required cell density: 6-cm plates for siRNA transfection assays, 24-well plates for IL-8/CXCL8 enzyme-linked immunosorbent assay (ELISA), and 10-cm plates for ChIP and coimmunoprecipitation assays.

### Transfection assay

Transfection was performed using Lipofectamine 3000, following the manufacturer’s instructions [27]. A549 cells were seeded in 6-cm plates and incubated overnight at 37 °C under 5% CO_2_ to allow for adherence. On the following day, scrambled and specific siRNAs were incubated with Lipofectamine 3000 in Opti-MEMat room temperature for 30 min. Meanwhile, the culture medium of A549 cells was replaced with serum-free DMEM/F12. Subsequently, the transfection mixture was added to the cells and incubated for 24 h. After transfection, the cells were treated with thrombin (10 U/mL) for the indicated periods. Cytoplasmic fractions and culture media were collected for analysis of IL-8/CXCL8 release and target protein inhibition.

### ELISA

An IL-8/CXCL8 assay was performed as described previously [27]. In brief, the culture media from BEAS-2B and A549 cells were collected, and IL-8/CXCL8 levels were measured using a commercially available ELISA kit (R&D Systems, Minneapolis, MN, USA).

### Coimmunoprecipitation assay

For coimmunoprecipitation assay, A549 cells were seeded in 10-cm plates and treated with thrombin (10 U/mL) for 10 min. After, the cells were lysed in 100 µL of immunoprecipitation lysis buffer containing protease and phosphatase inhibitors. Cell lysates were transferred to 1.5-mL microcentrifuge tubes and centrifuged at 8000 rpm for 30 min at 4 °C. The supernatants were incubated overnight at 4 °C with 5 µL of anti-ERK or anti-STK33 antibodies. The following day, the supernatants were incubated with protein A/G sepharose beads for 1 h at 4 °C. After incubation, the beads were collected through magnetic separation, and the supernatants were discarded. Finally, immunoprecipitated complexes were resuspended in 10 µL of 2.5X loading dye and boiled for Western blotting.

### Western blotting

Western blotting was performed as described previously [27]. BEAS-2B and A549 cells were cultured in 6-cm plates. After cell lysis and protein extraction, the extracted proteins were separated through sodium dodecyl sulfate polyacrylamide gel electrophoresis. The resultant protein bands were transferred onto polyvinylidene difluoride membranes. Subsequently, the membranes were blocked with 5% bovine serum albumin, washed, and incubated with specific primary antibodies. After washing, the membranes were incubated with horseradish peroxidase–conjugated secondary antibodies. Immunoreactivity was detected using an enhanced chemiluminescence reagent. Signal intensity was quantified using ImagePro (Eastman Kodak, Rochester, NY, USA).

### Real-time quantitative polymerase chain reaction (RT-qPCR)

Total RNA was extracted using Nucleozol (Macherey-Nagel) according to the manufacturer’s instructions. RNA concentration and purity were measured spectrophotometrically (NanoDrop^®^ ND-1000, Thermo Scientific). Complementary DNA (cDNA) was synthesized from total RNA using a reverse transcription kit. Diluted cDNA was amplified with gene-specific primers and SYBR Green Master Mix (Bio-Rad, Hercules, CA, USA). Quantitative PCR was performed using a Rotor-Gene Q Detection System (Qiagen, Chatsworth, CA, USA). Each sample was analyzed in triplicate. Relative gene expression was calculated using the ΔΔCt method, with β-actin serving as the internal control. The primers used were: human IL-8/CXCL8 (forward, 5′- GAGAGTGATTGAGAGTGGACCAC-3′; reverse, 5′- CACAACCCTCTGCACCCAGTTT-3′) and human β-actin (forward, 5′- CACCATTGGCAATGAGCGGTTC-3′; reverse, 5′- AGGTCTTTGCGGATGTCCACGT-3′).

### Chip assay

A549 cells were stimulated with thrombin (10 U/mL) for 30 min. The cells were then fixed with 10% formaldehyde for 10 min, harvested, and sonicated. Cell lysates were transferred to 1.5-mL microcentrifuge tubes and further sonicated to shear chromatin. Subsequently, the samples were centrifuged at 8000 rpm for 30 min at 4 °C, and the supernatants were subjected to immunoprecipitation with anti-c-Myc antibodies. Normal mouse IgG antibody was used as a negative control. The total DNA content in the lysates before immunoprecipitation was used for normalization. Precipitated DNA was purified and subjected to polymerase chain reaction with primers targeting the IL-8/CXCL8 promoter region (forward: 5′-ATAAAGTTATCTAGAAATAA-3′; reverse: 5′-TAATTCCAGTGGAGGCAT-3′). Amplicons were analyzed through 2% agarose gel electrophoresis to assess the binding of c-Myc to the IL-8/CXCL8 promoter.

### Immunohistochemistry

Airway tissue slides were deparaffinized using NovaHisto (20X diluted to 1X with double-distilled water) and heated in a pressure cooker (Cuisinart EPC-1,200) for 15 min. Subsequently, the slides were immersed in double-distilled water for 2 min. Tissue areas were outlined with a Pap waterproof pen, and sections were kept moist with 1X Tris-buffered saline with Tween-20 (TBST). Afterward, the tissue sections were incubated overnight at 4 °C with primary antibodies. The next day, the sections were washed thrice with 1X TBST, incubated with secondary antibodies for 30 min, and washed thrice with 1X TBST. After, the sections were incubated with 3,3′-diaminobenzidine substrate solution for color development, washed thrice with 1X TBST, and counterstained with hematoxylin for nuclear visualization. Finally, the stained sections were mounted with mounting medium for microscopic analysis.

### Immunofluorescence staining

Paraffin-embedded airway tissue sections were permeabilized with Triton X-100 for 10 min and blocked with 5% bovine serum albumin for 30 min at room temperature. Afterward, the sections were incubated with primary antibody for 1 h and then washed thrice with PBS. Next, the sections were incubated with Alexa Fluor 488- and Alexa Fluor 555-labeled secondary antibodies for 1 h and then washed thrice with PBS with Tween-20. Finally, the sections were mounted with 4′,6-diamidino-2-phenylindole-containing mounting medium and sealed with transparent nail polish. Target protein expression was visualized through immunofluorescence microscopy.

### Ethics approval

The use of human tissue samples was approved by the Joint Institutional Review Board of Taipei Medical University and its affiliated hospitals (approval numbers: N201702033, and N202405099). All animal protocols were approved by the Animal Ethics Committee of Taipei Medical University (approval number: LAC-2019-0360).

### Statistical analysis

Data are presented as mean ± standard error of the mean values. Cellular data were analyzed using unpaired one-way analysis of variance, followed by Dunnett’s test. The results of the ChIP and coimmunoprecipitation assays were analyzed using the unpaired Student *t* test. Data obtained from the mouse and patient tissue analyses are presented as mean ± standard deviation values and were compared using the unpaired Student *t* test with Dunnett’s test. A *p* value of < 0.05 was considered significant.

## Results

### STK33 expression was upregulated in airway epithelial cells from patients with severe asthma

**Fig. 1:**
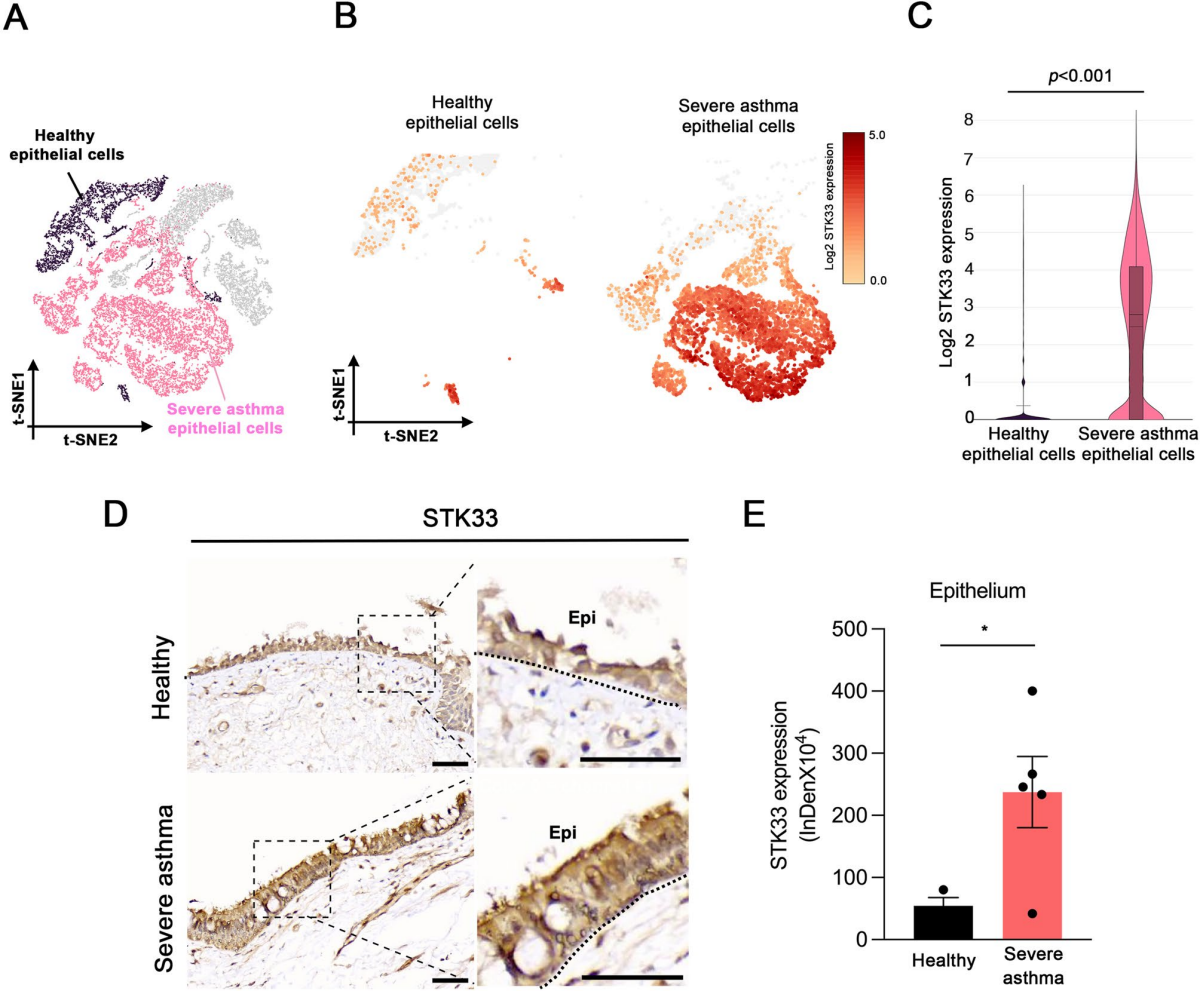
STK33 expression is upregulated in the airway epithelial cells of patients with severe asthma. **A**, t-distributed stochastic neighbor embedding revealing differences in epithelial cell clustering between healthy individuals (black) and patients with severe asthma (pink) **B**, Feature plots generated on the basis of single-cell RNA sequencing results depicting the levels of *STK33* expression in healthy and asthmatic airway epithelial cells. Darker red indicates higher expression levels **C**, Violin plot depicting the levels of *STK33* expression in healthy and asthmatic airway epithelial cells; *p < 0.05, compared with healthy cells **D**, Immunohistochemical staining of STK33 (brown) in bronchial biopsies from healthy individuals (*n* = 3) and patients with severe asthma (*n* = 5). Original magnification, 20× **E**, Levels of STK33 in airway epithelial cells from healthy individuals and patients with asthma. Data are presented as mean ± standard error of the mean values; *p < 0.05, compared with healthy individuals

We performed scRNA-seq to characterize epithelial cells from patients with severe asthma and healthy individuals. The results of t-distributed stochastic neighbor embedding indicated prominent differences in cell clustering between the two cohorts (Fig. 1A). STK33 expression was significantly higher in patients with asthma than in healthy individuals (Fig. 1B, C). To validate these findings, we performed immunohistochemical staining, which confirmed significantly higher STK33 expression in the airway epithelial cells of patients with severe asthma than in those of healthy individuals (Fig. 1D, E).

### Expression of STK33 and IL-8/CXCL8 (MIP-2) was upregulated in the mouse model of allergen-induced asthma

**Fig. 2:**
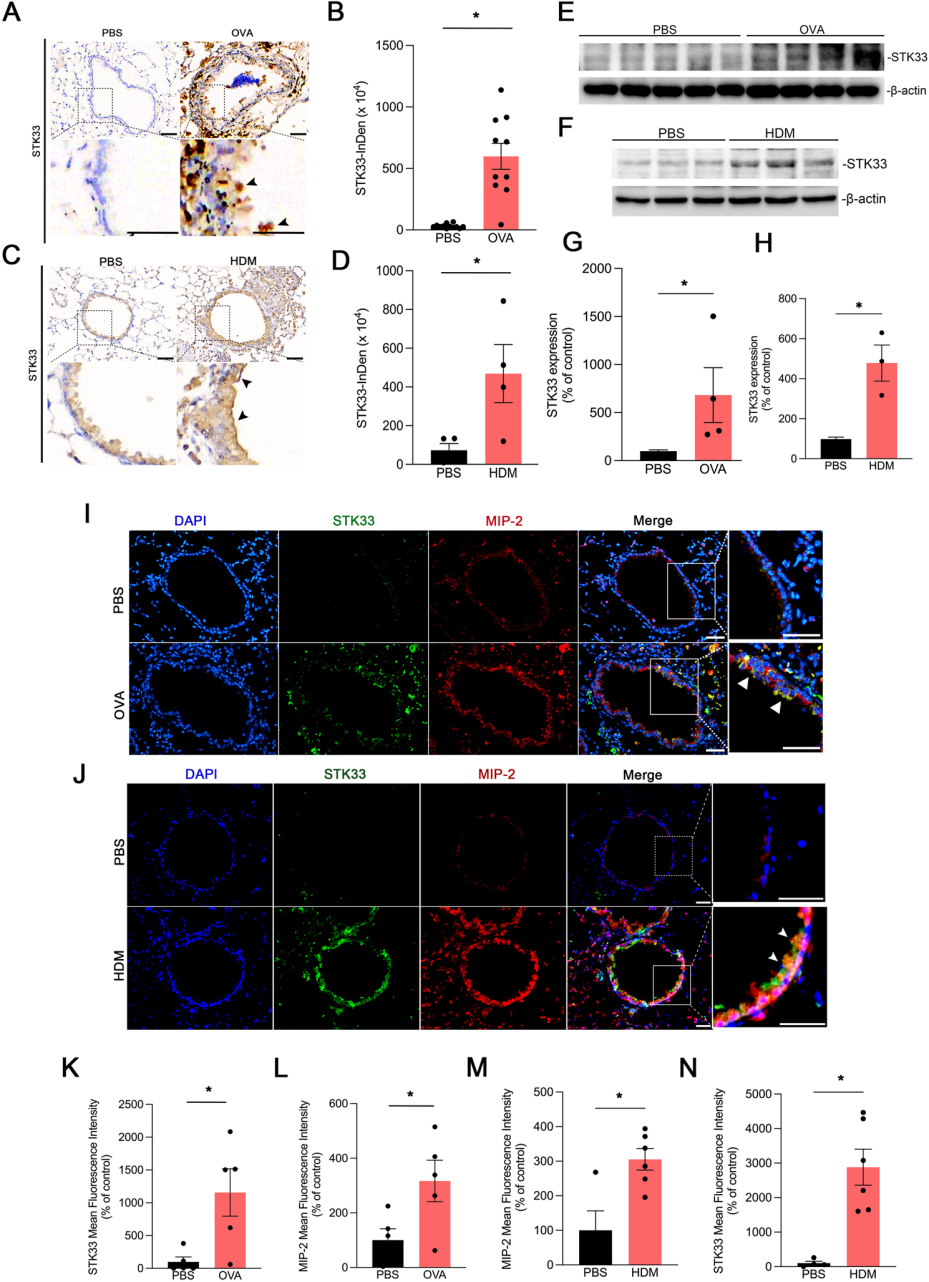
Expression of STK33 and MIP-2 (IL-8/CXCL8) is upregulated in the airway epithelial cells of ovalbumin- and HDM-challenged mice.**A**, Immunohistochemical staining of STK33 (brown) in lung tissues from PBS-treated and ovalbumin-challenged mice. Arrowheads indicate upregulated *STK33* expression in airway epithelial cells. Original magnification, 20× **B**, Levels of STK33 in PBS-treated mice (*n* = 12) and ovalbumin-challenged mice (*n* = 9), evaluated on the basis of integrated density. Data are presented as mean ± SEM values; *p < 0.05 **C**, Immunohistochemical staining of STK33 in lung tissues from PBS-treated and HDM-challenged mice. Arrowheads indicate upregulated *STK33* expression in airway epithelial cells. Original magnification, 20× **D**, Levels of STK33 in PBS-treated mice (*n* = 4) and HDM-challenged mice (*n* = 4), evaluated on the basis of integrated density. Data are presented as mean ± SEM values; *p < 0.05 Western blots depicting higher STK33 levels in the lung tissues of **(E)** ovalbumin- and **(F)** HDM-challenged mice than in those of PBS controls Densitometric quantification of STK33 normalized to β-actin in **(G)** ovalbumin-challenged mice (ovalbumin, *n* = 4; PBS, *n* = 5) and **(H)** HDM-challenged mice (HDM, *n* = 3; PBS, *n* = 3). Data are presented as mean ± SEM values; *p < 0.05 Immunofluorescence staining of lung tissues from **(I)** ovalbumin- and **(J)** HDM-challenged mice and PBS controls. The tissues were stained for STK33 (green), MIP-2 (red), and nuclei (4′,6-diamidino-2-phenylindole; blue). Merged images depict colocalization of STK33 and MIP-2 (arrowheads). Original magnification, 20× Mean fluorescence intensity of STK33 **(K)** and MIP-2 **(L)**, calculated from the images presented in Panel I. Data are presented as mean ± SEM values; *p < 0.05 (PBS, *n* = 5; ovalbumin, *n* = 5) Mean fluorescence intensity of STK33 **(M)** and MIP-2 **(N**), calculated from the images presented in Panel J. Data are presented as mean ± SEM values; *p < 0.05, compared with PBS controls (PBS, *n* = 5; HDM, *n* = 6)

We investigated the correlation of *STK33* expression with airway inflammation in mouse models of ovalbumin- and house dust mite (HDM)-induced asthma. Immunohistochemical staining revealed significantly higher STK33 levels in the airway epithelial cells of ovalbumin-challenged mice than in those of PBS-treated mice (control; Fig. 2A, B). Additionally, HDM-challenged mice exhibited markedly higher STK33 levels than did PBS controls (Fig. 2C, D). Western blotting further confirmed elevated STK33 levels in both ovalbumin- and HDM-challenged mice (Fig. 2E–H). To confirm the correlation between *STK33* expression and airway inflammation, we performed immunofluorescence staining. The expression of STK33 and IL-8/CXCL8 (murine homolog: MIP-2) was significantly upregulated in the airway tissues of both HDM- and ovalbumin-challenged mice (Fig. 2I, J). Quantitative analysis confirmed that the mean fluorescence intensity levels of both STK33 and MIP-2 were significantly higher in ovalbumin- and HDM-challenged mice than in PBS controls (Fig. 2K–N). Together, these findings indicate that upregulated STK33 expression is positively correlated with airway inflammation in allergen-induced asthma.

### STK33 mediates thrombin-induced IL-8/CXCL8 release from A549 and BEAS-2B cells

**Fig. 3:**
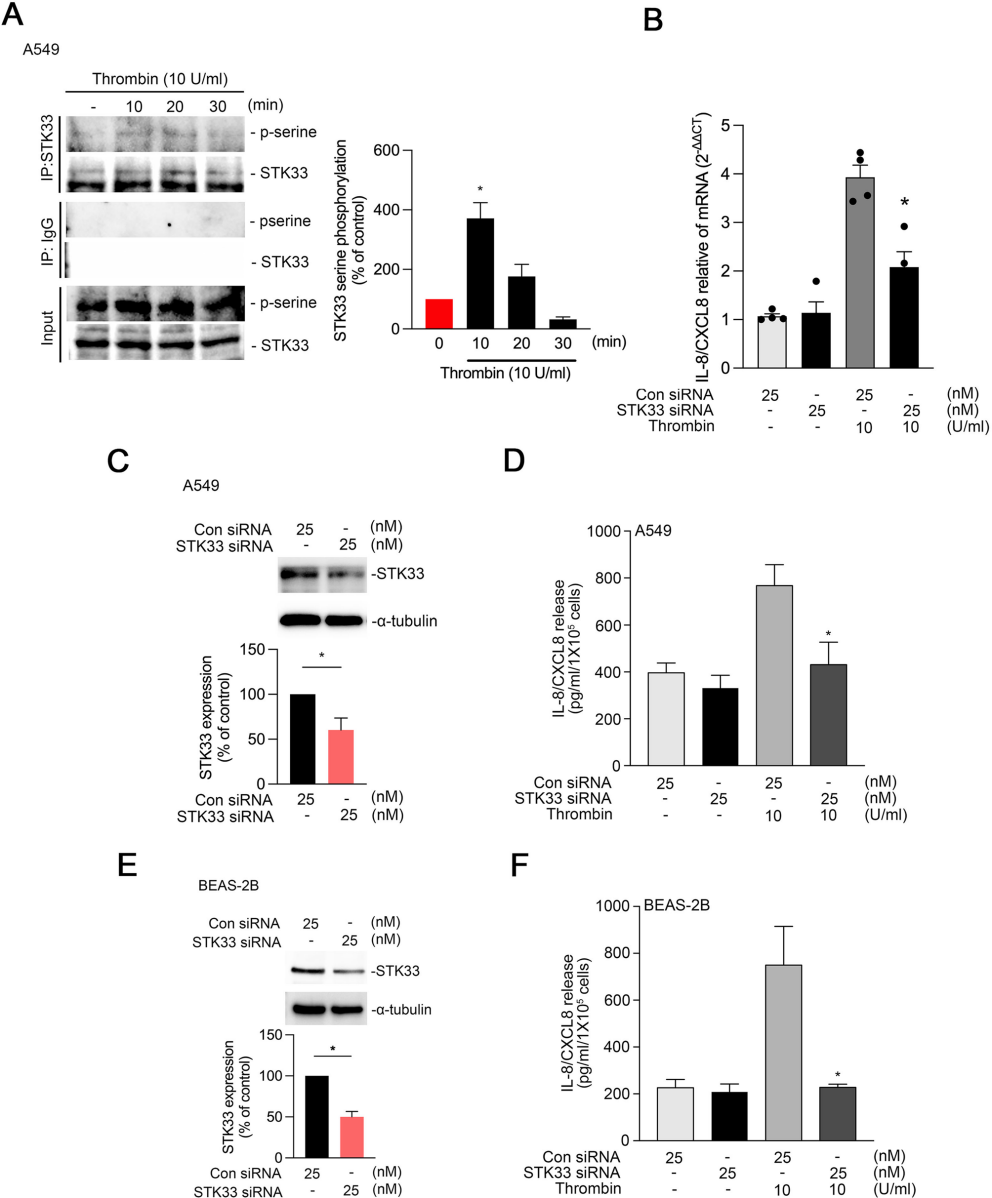
STK33 regulates thrombin-induced IL-8/CXCL8 expression in human lung epithelial cells.**A**, A549 cells were treated with thrombin (10 U/mL) for 0–30 min. Cell lysates were immunoprecipitated with anti-STK33 antibodies and immunoblotted with anti-phosphoserine and anti-STK33 antibodies. IgG was used as a negative control. Input samples represent total protein lysates. The bars present mean ± SEM values from four independent experiments; *p < 0.05, compared with untreated cells **B**, A549 cells were transfected with STK33 siRNA (25 nM) and then treated with thrombin. Total RNA was collected, and IL-8/CXCL8 mRNA levels were measured using qPCR. Data are presented as the mean ± S.E.M. (*n* = 4). * *p* < 0.05 compared with the PBS-treated group **C**, A549 cell lysates were immunoblotted with antibodies against STK33 and α-tubulin. The bars present mean ± SEM values from three independent experiments. *p < 0.05, compared with thrombin-treated wild-type cells **D**, A549 cells were transfected with scrambled or STK33 siRNA (25 nM) for 24 h and then treated with thrombin (10 U/mL) for another 24 h. The level of IL-8/CXCL8release was measured through ELISA **E**, BEAS-2B cells were transfected with scrambled or STK33 siRNA (25 nM) for 24 h ours, then treated with thrombin (10 U/mL) for an additional 24 h. The IL-8/CXCL8 release level was measured using ELISA **F**, BEAS-2B cell lysates were immunoblotted with antibodies against STK33 and α-tubulin. The bars show mean ± SEM values from three independent experiments; *p < 0.05, compared with thrombin-treated wild-type cells

To elucidate the role of STK33 in thrombin-induced IL-8/CXCL8 release, we first investigated STK33 phosphorylation in thrombin-treated A549 cells. For this, we performed immunoprecipitation with an STK33 antibody, followed by Western blotting. The results revealed increased serine phosphorylation of STK33 at 10 min after thrombin treatment (Fig. 3A). STK33 siRNA down-regulated thrombin-induced IL-8/CXCL8 mRNA level in A549 cells (Fig. 3B). Furthermore, ELISA indicated that treatment with STK33 siRNA significantly reduced thrombin-induced IL-8/CXCL8 release by 73.06% ± 25.92% in A549 cells and by 96.33% ± 2.65% in BEAS-2B cells (Fig. 3C–F).

### STK33 interacts with ERK in thrombin-treated human lung epithelial cells

**Fig. 4:**
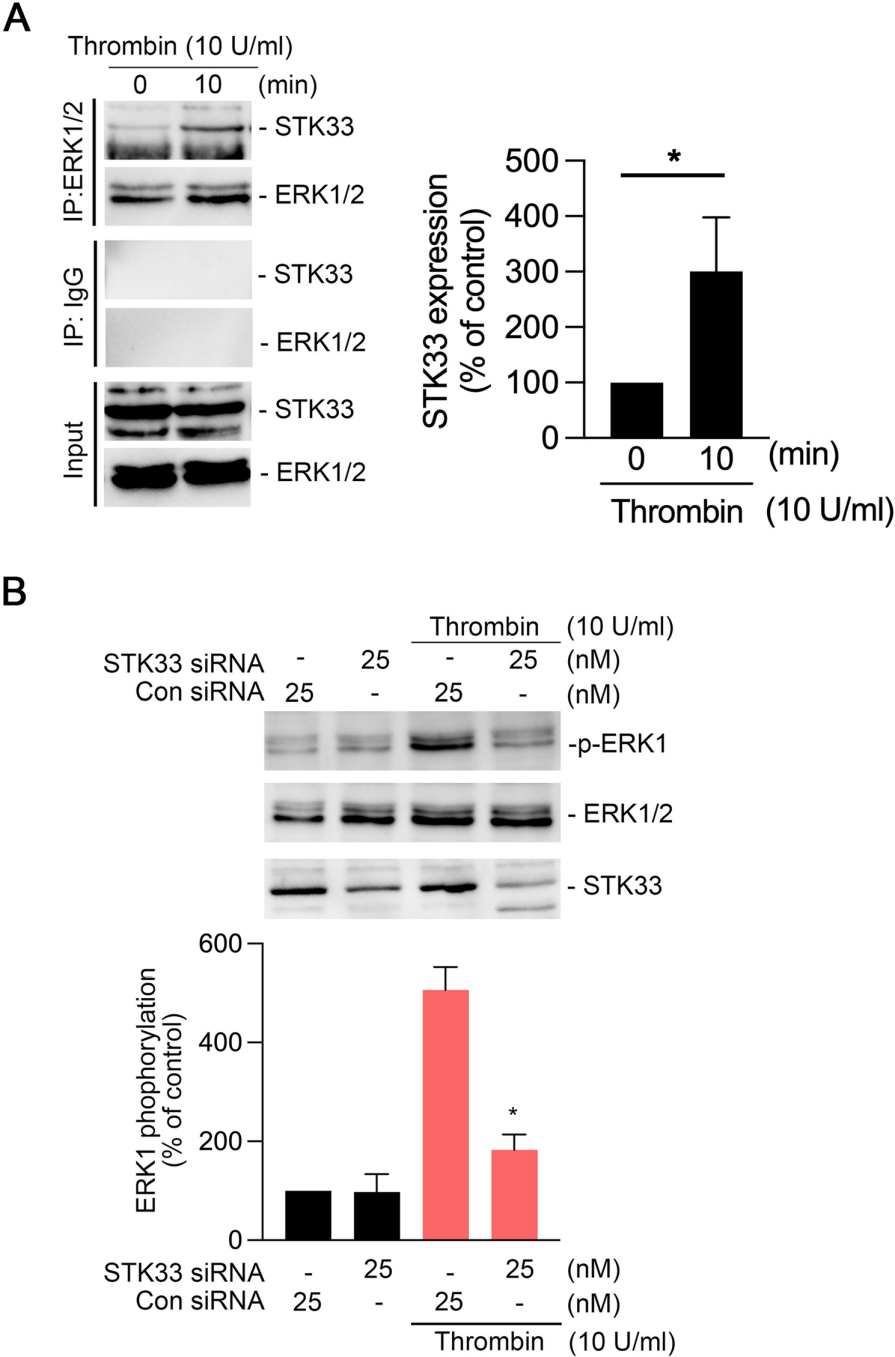
STK33 interacts with ERK and regulates thrombin-induced ERK phosphorylation.**A**, A549 cells were treated with thrombin (10 U/mL) for 10 min. After, the cells were immunoprecipitated with antibodies against ERK1/2 and subsequently immunoblotted with antibodies against STK33 and ERK1/2 to enable investigation of the formation of the ERK1/2–STK33 complex. IgG was used as a negative control. Input samples represent total protein lysates. Data are presented as mean ± SEM values from four independent experiments; *p < 0.05, compared with untreated cells **B**, A549 cells were transfected with scrambled or STK33 siRNA for 24 h and then treated with thrombin (10 U/mL) for 10 min. Cell lysates were immunoblotted with antibodies against phosphorylated ERK1, total ERK1/2, and STK33. Data are presented as mean ± SEM values from three independent experiments; *p < 0.05, compared with thrombin-treated wild-type cells

A Previous study have indicated that ERKs can bind to STK33 and promote ERK activation in 293 T cells [28]. To examine whether a similar interaction occurs in the thrombin signaling pathway, we next investigated the potential interaction between STK33 and ERK in thrombin-treated A549 cells. Immunoprecipitation with an ERK1/2 antibody revealed increased binding between STK33 and ERK1/2 after thrombin treatment (Fig. 4A). Treatment with STK33 siRNA (25 nM) significantly reduced thrombin-induced ERK1 phosphorylation in A549 cells (Fig. 4B). These findings suggest that STK33 interacts with ERK1/2 and thus mediates thrombin-induced ERK activation in lung epithelial cells.

### ERK–STK33 complex mediates thrombin-induced c-Myc phosphorylation in airway inflammation

**Fig. 5:**
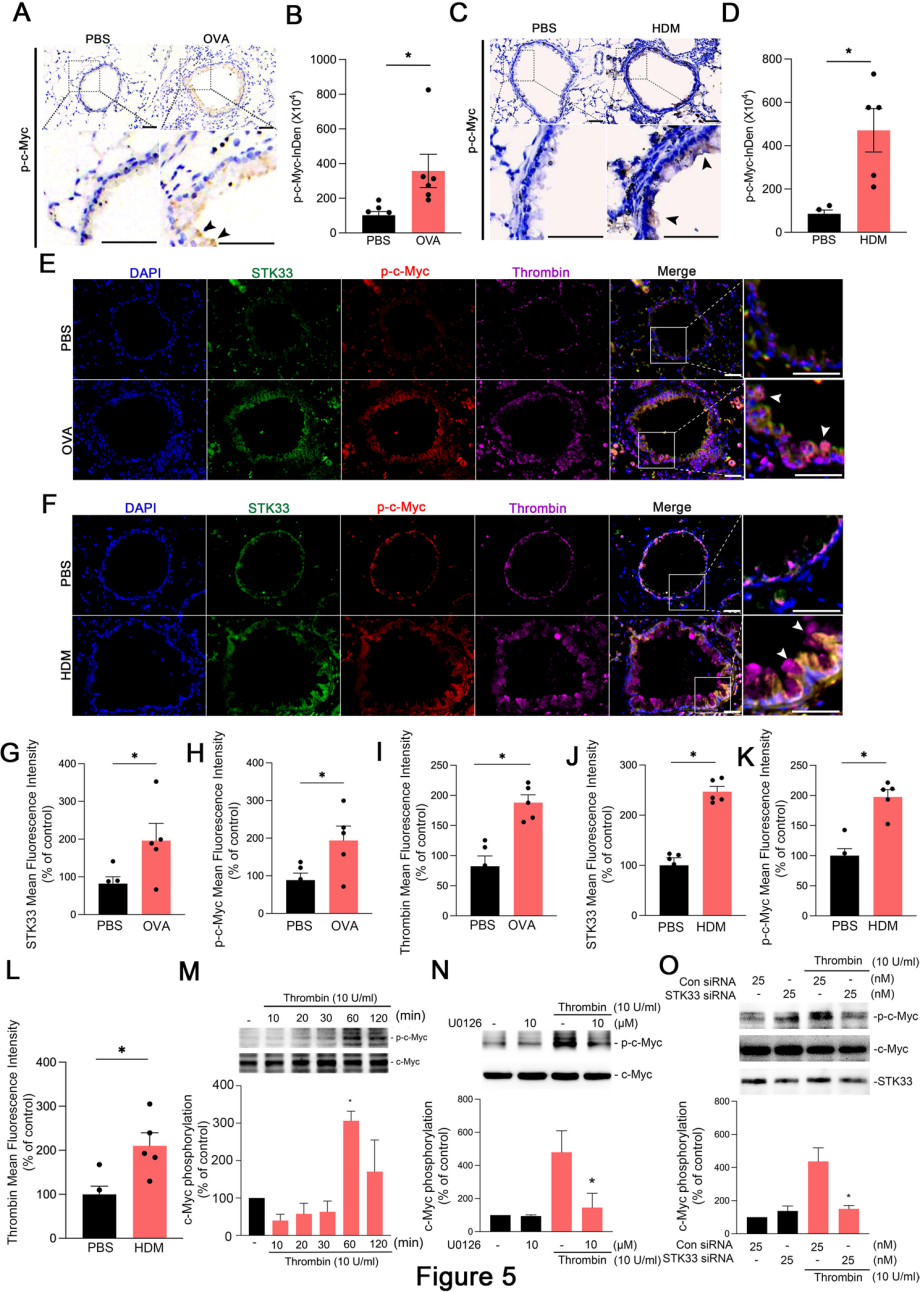
Thrombin induces c-Myc phosphorylation in A549 cells and asthmatic mice.**A**, Immunohistochemical staining of phosphorylated c-Myc in lung tissues from PBS-treated (control) and ovalbumin-challenged mice. Original magnification, 20× **B**, Level of phosphorylated c-Myc in airway tissues from PBS-treated mice (*n* = 6) and ovalbumin-challenged mice (*n* = 6), evaluated on the basis of integrated density. Data are presented as mean ± SEM values; *p < 0.05 **C**, Immunohistochemical staining of phosphorylated c-Myc in lung tissues from PBS-treated and HDM-challenged mice. Original magnification, 20× **D**, Levels of phosphorylated c-Myc in PBS-treated mice (*n* = 5) and HDM-challenged mice (*n* = 5), evaluated on the basis of integrated density. Data are presented as mean ± SEM values; *p < 0.05 **E**, Representative triple immunofluorescence staining for STK33 (green), phosphorylated c-Myc (p-c-Myc; red), and thrombin (magenta) in lung tissues from PBS- and OVA-challenged mice (*n* = 5, original magnification ×20). Nuclei were counterstained with DAPI (blue). Quantification of mean fluorescence intensity (MFI) of **(G)** STK33, **(H)** p-c-Myc, and **(I)** thrombin in PBS- and OVA-challenged mice using ImageJ software. Data are presented as mean ± SEM. *p < 0.05 compared with the PBS-treated group **F**, Representative triple immunofluorescence staining for STK33 (green), p-c-Myc (red), and thrombin (magenta) in lung tissues from PBS- and HDM-challenged mice (*n* = 5, original magnification ×20). Nuclei were counterstained with DAPI (blue). Quantification of MFI of **(J)** STK33, **(K)** p-c-Myc, and **(L)** thrombin in PBS- and HDM-challenged mice using ImageJ software. Data are presented as mean ± SEM. *p < 0.05 compared with the PBS-treated group **M**, A549 cells were treated with thrombin (10 U/mL) for 0–120 min. Cell lysates were immunoblotted with antibodies against c-Myc or phosphorylated c-Myc. The bars present mean ± SEM values from three independent experiments; *p < 0.05, compared with untreated cells **N**, A549 cells were incubated with U0126 for 30 min and then treated with thrombin for 60 min. Cell lysates were immunoblotted with antibodies against c-Myc or phosphorylated c-Myc. The bars present mean ± SEM values from three independent experiments; *p < 0.05, compared with cells treated with thrombin alone **O**, A549 cells were transfected with scrambled or STK33 siRNA (25 nM) for 24 h and then treated with thrombin (10 U/mL) for 60 min. Cell lysates were immunoblotted with antibodies against c-Myc or phosphorylated c-Myc. The bars present mean ± SEM values from three independent experiments; *p < 0.05, compared with thrombin-treated wild-type cells

A previous study showed that STK33 enhances the transcriptional activity of c-Myc, thereby regulating the proliferation and differentiation of hepatocellular carcinoma cells [23]. Next, In the present study, we next aimed to measure the levels of thrombin, phosphorylated c-Myc, and STK33 expression in allergen-induced asthma, and to investigate their regulation by the ERK–STK33 complex in thrombin-induced airway inflammation. First, we evaluated c-Myc phosphorylation in a mouse model of allergen-induced asthma. Immunohistochemical staining revealed that the level of phosphorylated c-Myc was significantly higher in the airway epithelial cells of both ovalbumin- and HDM-challenged mice than in those of PBS controls (Fig. 5A–D). Triple immunofluorescence staining for STK33, p-c-Myc, and thrombin was performed on lung tissues from PBS-, OVA-, and HDM-challenged mice, revealing a colocalization increase in the expression of all three proteins in the OVA- and HDM-challenged groups compared with PBS controls (Fig. 5E–K). In human lung epithelial A549 cells, thrombin treatment markedly increased c-Myc phosphorylation, which peaked at 60 min after treatment (Fig. 5M). Notably, treatment with the ERK inhibitor U0126 markedly reduced the level of phosphorylation (Fig. 5N). Moreover, treatment with STK33 siRNA significantly reduced thrombin-induced c-Myc phosphorylation (Fig. 5O). These findings highlight the vital roles of ERK and STK33 in the thrombin-mediated activation of c-Myc in airway inflammation.

### c-Myc mediates thrombin-induced IL-8/CXCL8 expression in human lung epithelial cells

**Fig. 6:**
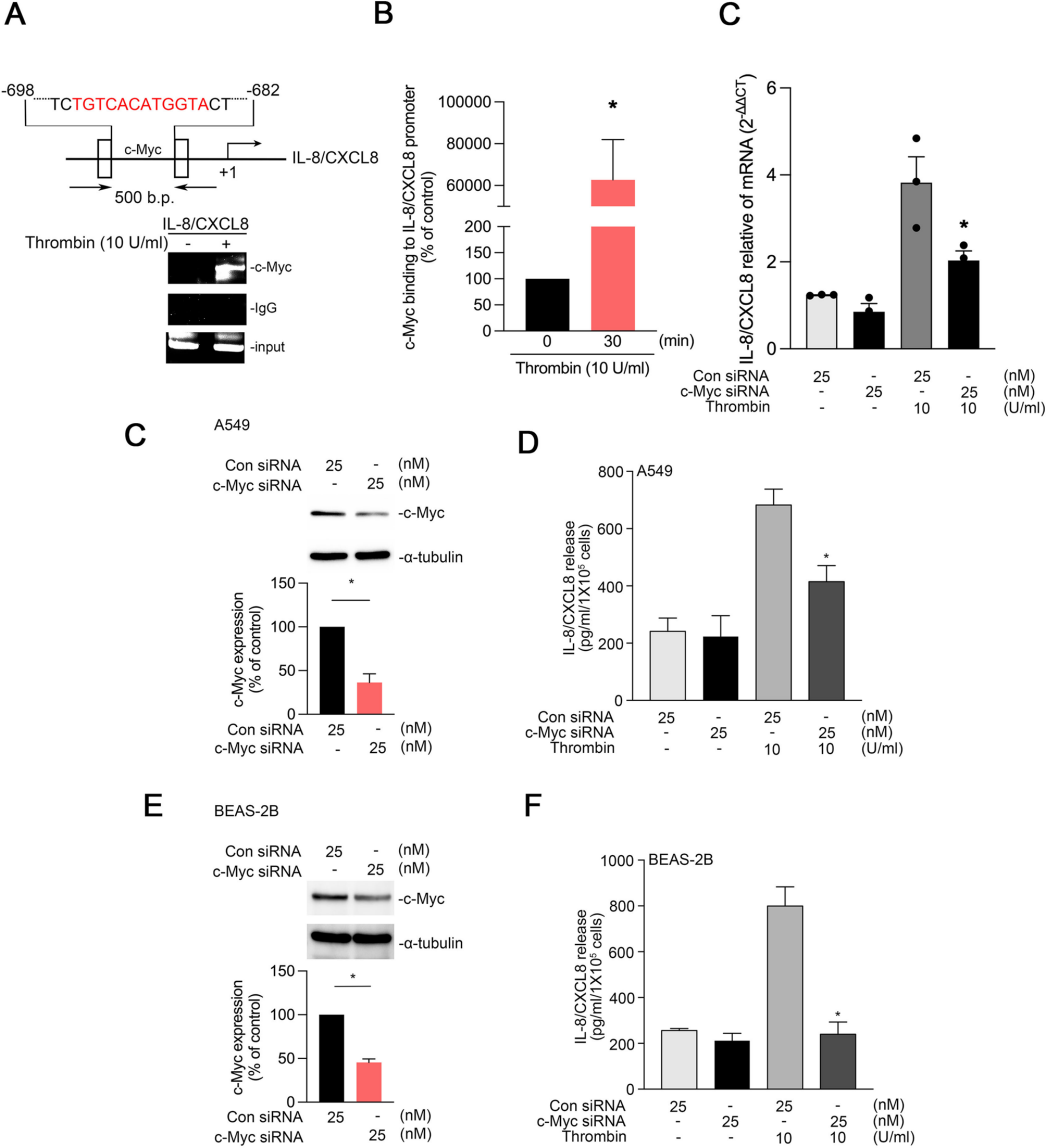
c-Myc mediates thrombin-induced IL-8/CXCL8 expression in human lung epithelial cells.**A**, A549 cells were treated with thrombin (10 U/mL) for 30 min. Cell lysates were subjected to ChIP analysis with antibodies against c-Myc to assess its binding to the IL-8/CXCL8 promoter. Input DNA was used as the positive control, and immunoglobulin G was used as the negative control (*n* = 3) **B**, ChIP assay results indicating increased c-Myc binding to the IL-8/CXCL8 promoter after thrombin treatment. Data are presented as mean ± SEM values (*n* = 3); *p < 0.05, compared with untreated cells **C**, A549 cells were transfected with c-Myc siRNA (25 nM) and then treated with thrombin. Total RNA was collected, and IL-8/CXCL8 mRNA levels were measured using qPCR. Data are presented as the mean ± S.E.M. (*n* = 3). * *p* < 0.05 compared with the PBS-treated group **D**, A549 cells were transfected with scrambled or c-Myc siRNA (25 nM) for 24 h. Cell lysates were immunoblotted with antibodies for c-Myc and α-tubulin **E**, A549 cells were transfected with scrambled or c-Myc siRNA (25 nM) for 24 h and then treated with thrombin (10 U/mL) for another 24 h. The level of IL-8 release was measured through ELISA. The bars present mean ± SEM values from three independent experiments. *p < 0.05, compared with thrombin-treated wild-type cells **F**, BEAS-2B cells were transfected with scrambled or c-Myc siRNA (25 nM) for 24 h. Cell lysates were immunoblotted with antibodies against c-Myc and α-tubulin. The bars present mean ± SEM values from three independent experiments; *p < 0.05, compared with thrombin-treated wild-type cells **G**, BEAS-2B cells were transfected with scrambled or c-Myc siRNA (25 nM) for 24 h and then treated with thrombin (10 U/mL) for another 24 h. The level of IL-8 release was measured through ELISA

To investigate whether c-Myc directly regulates IL-8/CXCL8 expression in human lung epithelial cells, we performed a ChIP assay. The results revealed that thrombin treatment significantly enhanced the binding of c-Myc to the IL-8/CXCL8 promoter (Fig. 6A, B). c-Myc siRNA decreased thrombin-induced IL-8/CXCL8 mRNA level in A549 cells (Fig. 6C) Consistent with this finding, ELISA indicated that treatment with c-Myc siRNA reduced thrombin-induced IL-8/CXCL8 release by approximately 56.70% ± 12.79% in A549 cells and 94.72% ± 9.76% in BEAS-2B cells (Fig. 6D, E). Western blotting confirmed effective knockdown of c-Myc expression after siRNA treatment (Fig. 6F, G).

## Discussion

IL-8/CXCL8 is a neutrophil-attracting chemokine and a key contributor to asthma exacerbation [29]. To the best of our knowledge, the present study is the first to elucidate the role of STK33 in thrombin-induced IL-8/CXCL8 release from human lung epithelial cells. We noted high levels of STK33 in airway epithelial cells from patients with severe asthma and those from ovalbumin- and HDM-challenged mice. Thrombin induced IL-8/CXCL8 release by activating the ERK/STK33 pathway, which subsequently induced the phosphorylation and nuclear translocation of c-Myc. Phosphorylated c-Myc bound to the IL-8/CXCL8 promoter. These findings indicate that STK33 mediates thrombin-induced IL-8/CXCL8 release from human lung epithelial cells. Thus, our study highlights the pathological role of STK33 in asthma.

Evidence suggests that STK33 is upregulated in various cancers, including prostate, lung, and gastric cancers, where it promotes tumor growth and angiogenesis [30]. STK33 exerts this effect by interacting with the HSP90 chaperone, thereby regulating the hypoxia-inducible factor 1α/vascular endothelial growth factor pathway [31, 32]. Furthermore, STK33 undergoes autophosphorylation and forms a complex with vimentin [14]. STK33 phosphorylates ERK2, enhancing its activity and promoting tumorigenesis, as observed in colorectal cancer cells (HCT15 cells) [28]. We discovered that thrombin induced the activation of STK33 and the formation of the ERKs–STK33 complex. Treatment with STK33 siRNA significantly reduced thrombin-induced ERK1 phosphorylation in human lung epithelial cells. This indicates that STK33 regulates ERK1 phosphorylation, thereby inducing IL-8/CXCL8 expression. Collectively, these findings implicate STK33 in cancer development (angiogenesis and tumor growth) and asthma progression. However, the mechanisms underlying the thrombin-induced expression of *STK33* remain to be elucidated.

STKs represent a diverse family of intracellular kinases that phosphorylate hydroxyl groups on serine or threonine residues of target proteins. Multiple STKs have been associated with cancer pathogenesis [33]. For instance, macrophage-stimulating protein 1 (STK4) and macrophage-stimulating protein 2 (STK3), major components of the Hippo pathway, regulate mucous metaplasia and club cell proliferation in airway tissues [34]. Specifically, STK4 is a key mediator of T-cell receptor–induced formation of the nuclear factor-κB p65–Foxp3 complex through Foxp3 phosphorylation at serine 418, with this modification essential for regulatory T cell–mediated immune tolerance [35]. In the present study, STK33, another member of the STK family, emerged as a vital regulator of thrombin-induced IL-8/CXCL8 release from human lung epithelial cells. Our results reveal that thrombin increased STK33 phosphorylation at serine residues, suggesting that the phosphorylation and subsequent activation of STK33 mediate thrombin-induced IL-8/CXCL8 release. These findings expand the understanding of STK family functions in airway inflammation and highlight them as potential therapeutic targets in inflammatory airway diseases.

Notably, c-Myc is a transcription factor that regulates cellular growth, differentiation, and apoptosis, playing a central role in cancer development [18–20]. A study reported that c-Myc knockdown markedly reduced *Alternaria*-induced airway inflammation and airway hyperresponsiveness [22]. c-Myc has been reported to promote the production of type 2 cytokines, including IL-5 and IL-13, in response to epithelial-derived cytokines such as TSLP, IL-33, and IL-25 [22]. However, whether c-Myc also regulates epithelial-derived cytokines in severe asthma remains unclear and needs further investigation. In the present study, thrombin increased c-Myc phosphorylation in A549 cells, and treatment with c-Myc siRNA significantly attenuated thrombin-induced IL-8/CXCL8 release. Our findings also indicate that thrombin promoted the binding of c-Myc to the IL-8/CXCL8 promoter, which supports the regulatory role of c-Myc in airway inflammation. Research indicates that STK33 enhances the transcriptional activity of c-Myc in hepatocellular carcinoma, highlighting a broader regulatory role of STK33 in c-Myc-dependent signaling [23]. Consistent with these findings, our study revealed upregulated c-Myc expression in airway epithelial cells from ovalbumin- and HDM-challenged mice. Treatment with STK33 siRNA significantly reduced thrombin-induced c-Myc phosphorylation. Collectively, these findings indicate that STK33-mediated activation of c-Myc is a key mechanism that drives thrombin-induced IL-8/CXCL8 expression in human lung epithelial cells.

This study has some limitations. First, the use of A549 cells cannot fully recapitulate the characteristics of airway epithelial cells in vivo. To address this concern, we also performed thrombin-induced IL-8/CXCL8 release experiments using ELISA in BEAS-2B cells, which showed similar responses, thereby supporting the relevance of our findings. Second, although our data suggest that STK33 is critically involved in thrombin-induced IL-8/CXCL8 expression, we did not perform in vivo experiments directly targeting STK33 inhibition in asthmatic mouse models. Such validation would be valuable for further supporting the therapeutic potential of targeting STK33 in airway inflammation. However, we observed increased co-expression of thrombin, STK33, and IL-8/CXCL8 in the lung tissues of asthmatic mice. It may be indicated that STK33 plays a role in thrombin-induced IL-8/CXCL8 expression in human airway epithelial cells.

**Fig. 7:**
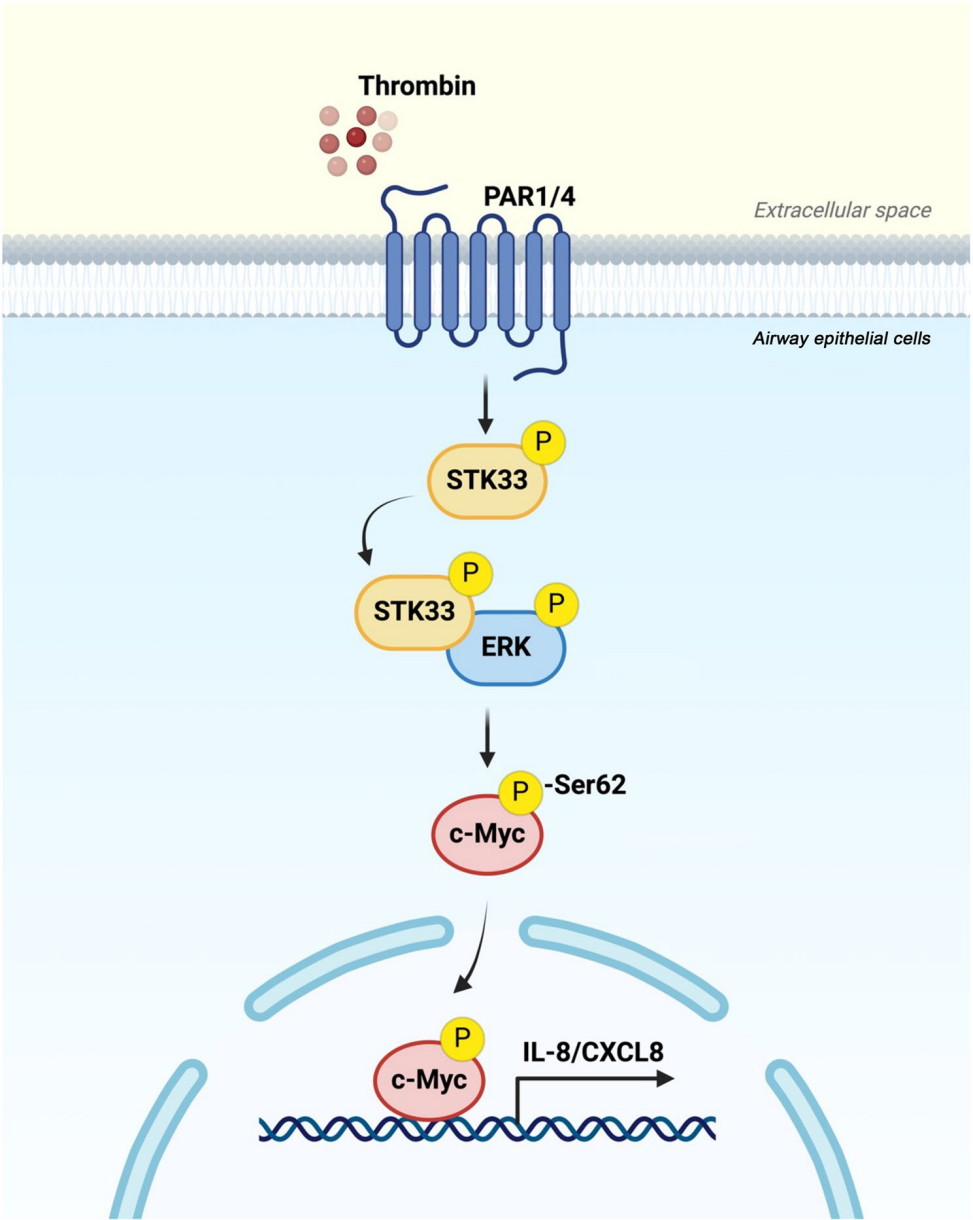
ERK/STK33/c-Myc signaling mediates thrombin-induced IL-8/CXCL8 expression in human lung epithelial cells. Thrombin treatment in A549 cells leads to STK33 phosphorylation and ERK–STK33 complex formation, thereby increasing c-Myc phosphorylation, IL-8/CXCL8 expression, and IL-8/CXCL8 release. The schematic was created using BioRender (https://biorender.com/)

We noted that STK33 was overexpressed in severe asthma. It facilitated thrombin-induced IL-8/CXCL8 release from human lung epithelial cells through the ERK/STK33/c-Myc pathway (Fig. 7). Thus, this study unveiled a previously unrecognized role of STK33 in asthma-related airway inflammation. Therefore, STK33 may be targeted for managing airway inflammation in patients with severe asthma.

## Supplementary Information

Supplementary Material 1.

## Data Availability

The single-cell RNA sequencing data of healthy control trachea tissue were obtained from the publicly available GEO dataset GSE134174 ([https://www.ncbi.nlm.nih.gov/geo/query/acc.cgi?acc=GSE134174](https:/www.ncbi.nlm.nih.gov/geo/query/acc.cgi?acc=GSE134174)). The processed Loupe Browser file (.loupe) generated in this study is available at Zenodo (DOI: 10.5281/zenodo.16870229). All other data supporting the findings of this study are available from the corresponding author upon reasonable request.

## Abbreviations

ChIP: Chromatin immunoprecipitation assay
DAPI: 4′,6-diamidino-2-phenylindole
DMEM/F-12: Dulbecco’s Modified Eagle Medium/Nutrient Mixture F-12
ERK: Extracellular signal-regulated kinase
HDM: House dust mite
IL-8/CXCL8: Interleukin-8
MIP-2: Macrophage inflammatory protein 2
PBS: Phosphate-buffered saline
scrambled siRNA: Control small interfering RNA
scRNA-seq: Single-cell RNA sequencing
STK: Serine/threonine kinase
TBST: Tris-buffered saline with Tween-20
