# Supplementary material for: Serine/threonine kinase 33 mediates thrombin-induced interleukin-8 release from human lung epithelial cells in severe asthma
Source: Respir Res. 2025 Oct 21;26:288. doi: 10.1186/s12931-025-03368-6 (PMC12539136; doi:10.1186/s12931-025-03368-6)
Supplement: Supplementary file 1 — Supplementary Material 1. [file 12931_2025_3368_MOESM1_ESM.pdf]

STK33 mediates thrombin-induced IL-8/CXCL8 release in human lung epithelial cells in severe asthma

WB RAW

Fig 1D

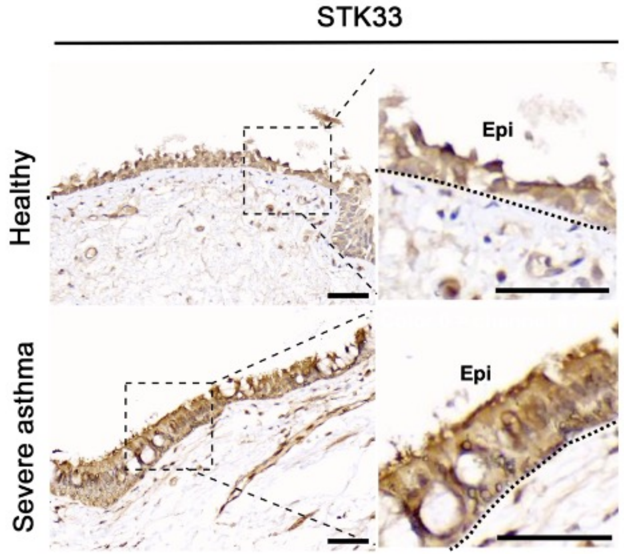
STK33
Healthy
Severe asthma
Epi
Epi

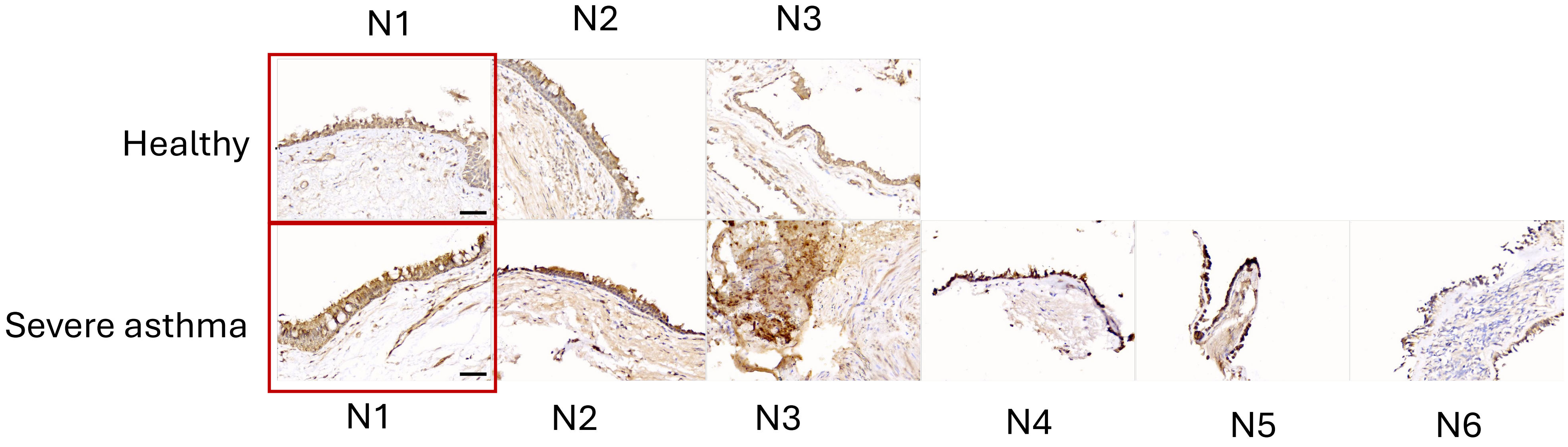
N1
N2
N3
Healthy
Severe asthma
N1
N2
N3
N4
N5
N6

Fig 2F

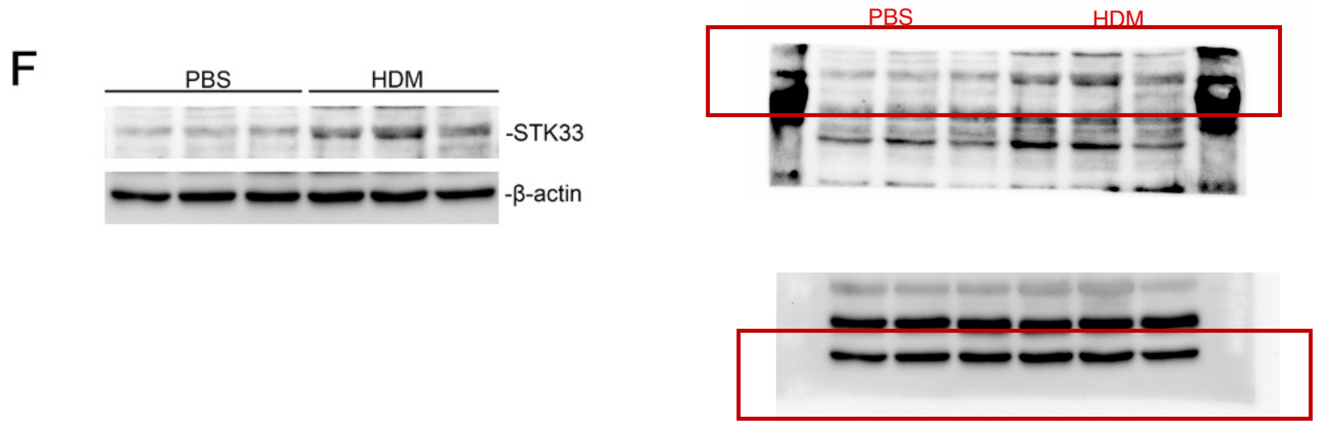

Fig 2E

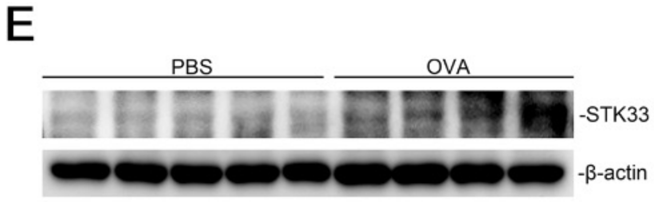

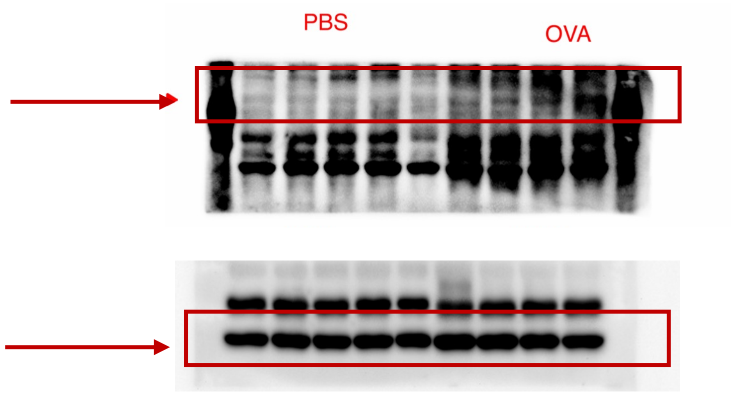

Fig 3A

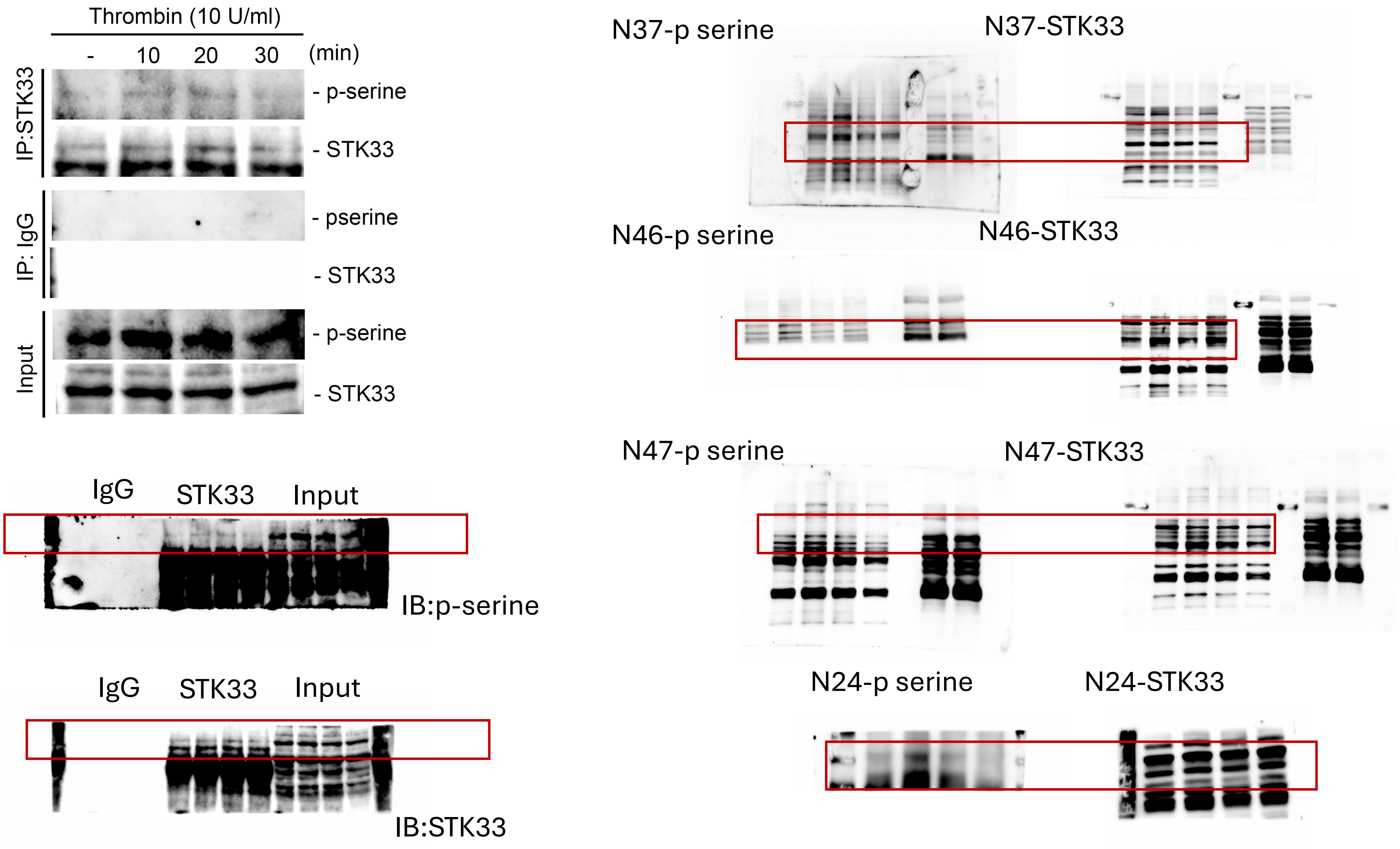

Thrombin (10 U/ml)
- 10 20 30 (min)
IP:STK33
- p-serine
- STK33
IP: IgG
- pserine
- STK33
Input
- p-serine
- STK33
IgG STK33 Input
IB:p-serine
IgG STK33 Input
IB:STK33
N37-p serine
N37-STK33
N46-p serine
N46-STK33
N47-p serine
N47-STK33
N24-p serine
N24-STK33

Fig 3C

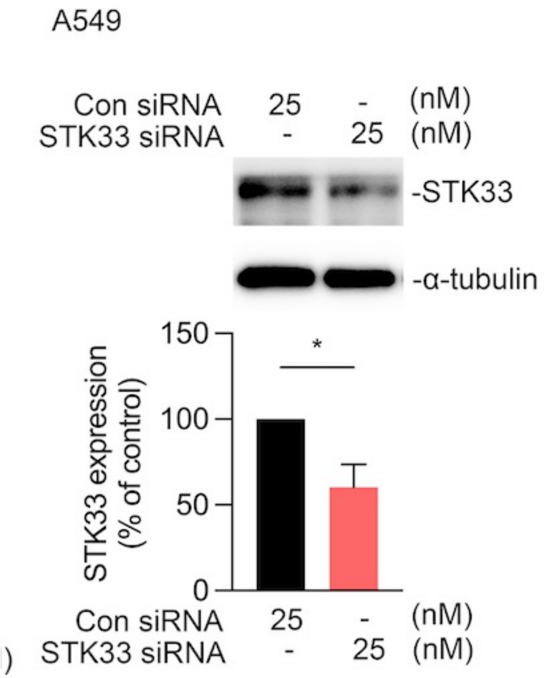

STK33 tub

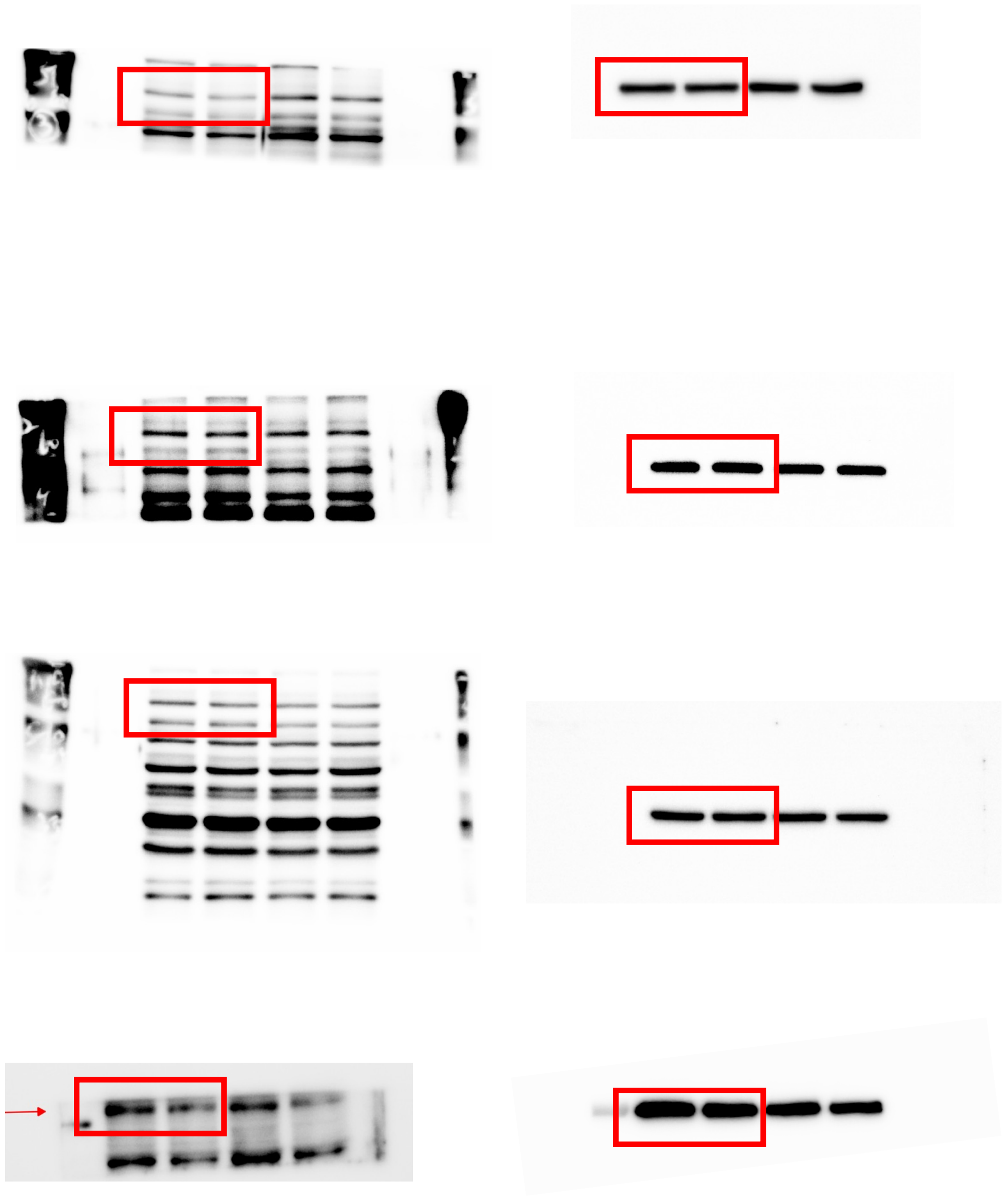

Fig 3E

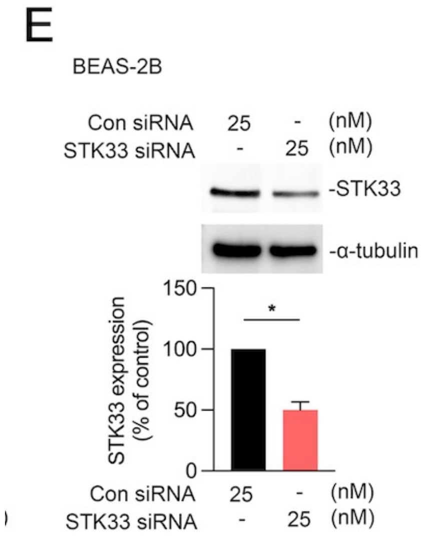

STK33 tub

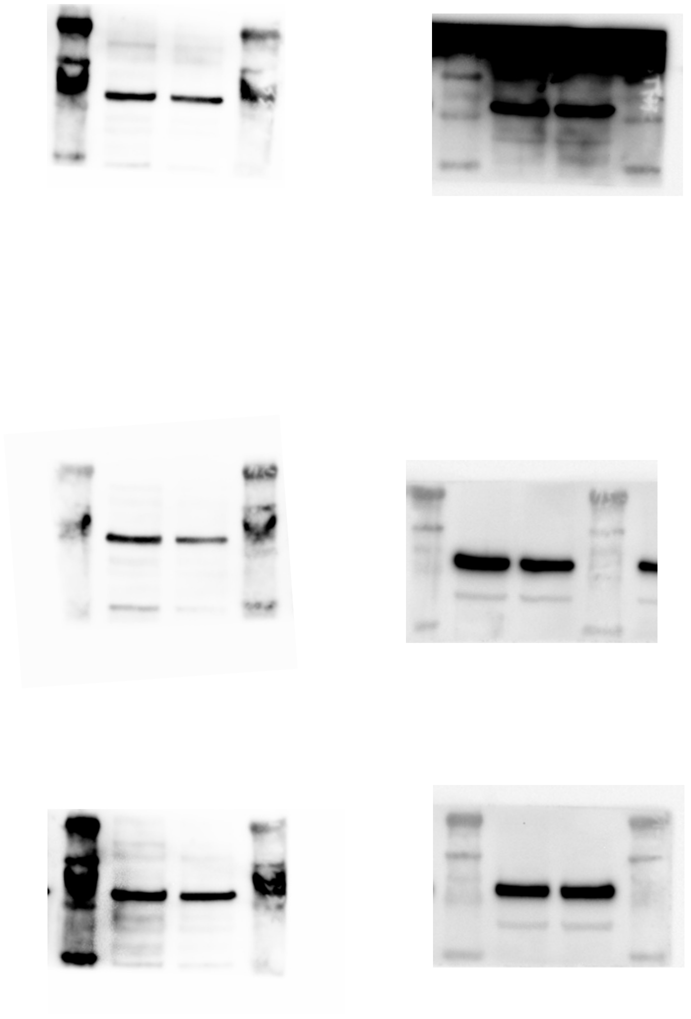

Fig 4A

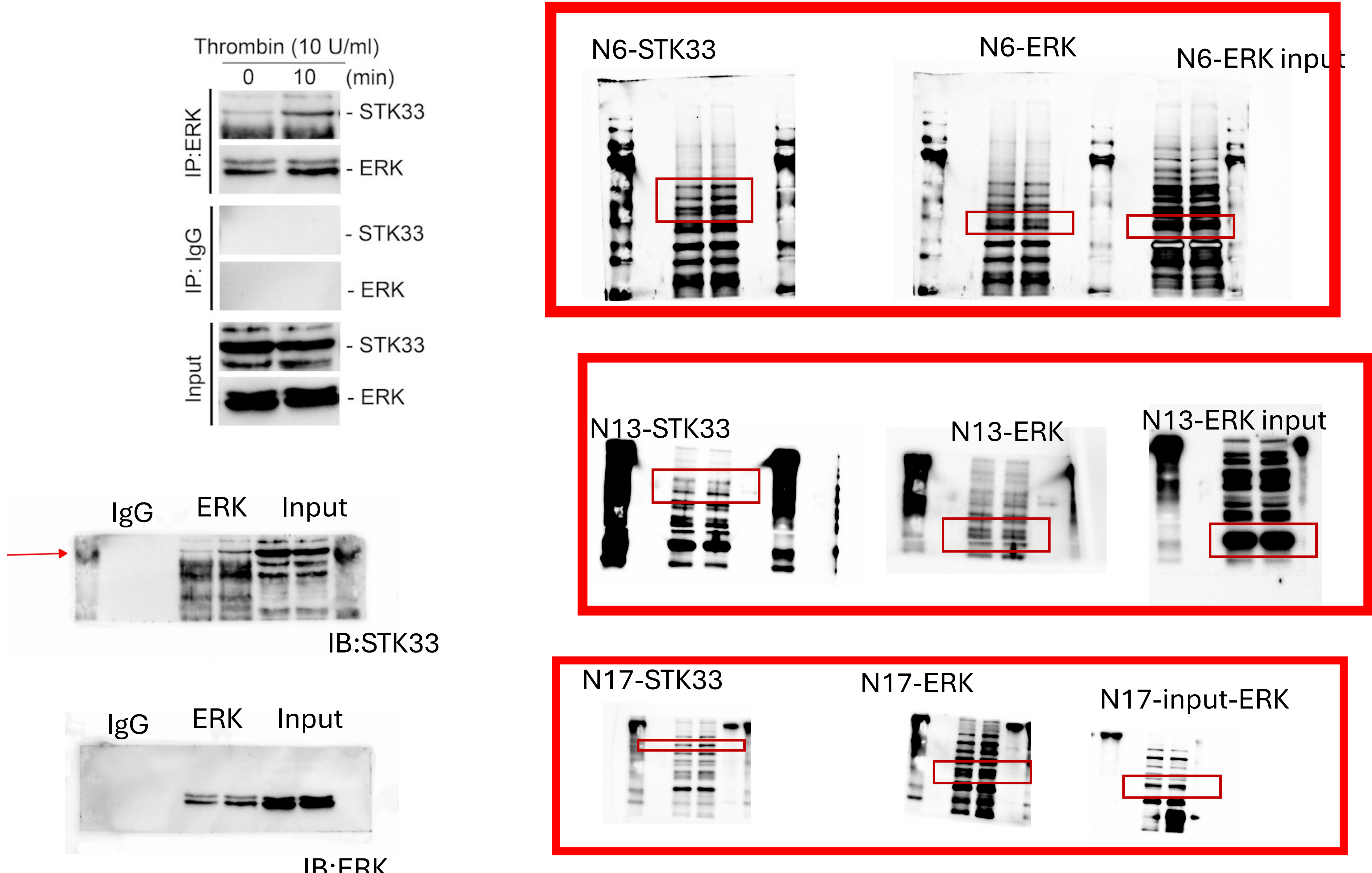
Thrombin (10 U/ml)
0
10
(min)
IP:ERK
- STK33
- ERK
IP: IgG
- STK33
- ERK
Input
- STK33
- ERK
IgG
ERK
Input
IB:STK33
IgG
ERK
Input
IB:ERK
N6-STK33
N6-ERK
N6-ERK input
N13-STK33
N13-ERK
N13-ERK input
N17-STK33
N17-ERK
N17-input-ERK

Fig 4B

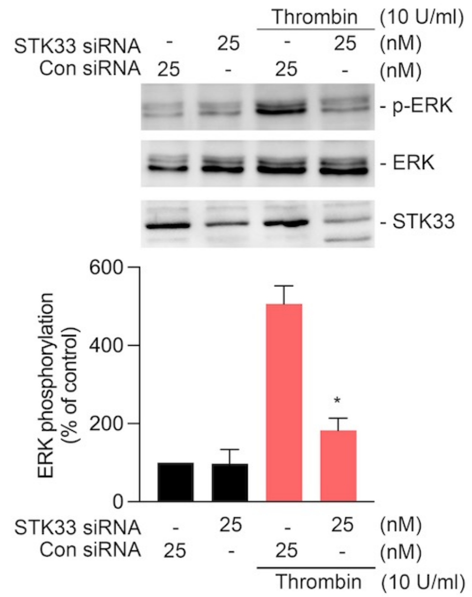
Thrombin (10 U/ml)
STK33 siRNA - 25 - 25 (nM)
Con siRNA 25 - 25 - (nM)
- p-ERK
- ERK
- STK33
ERK phosphorylation (% of control)
600
400
200
0
*
STK33 siRNA - 25 - 25 (nM)
Con siRNA 25 - 25 - (nM)
Thrombin (10 U/ml)

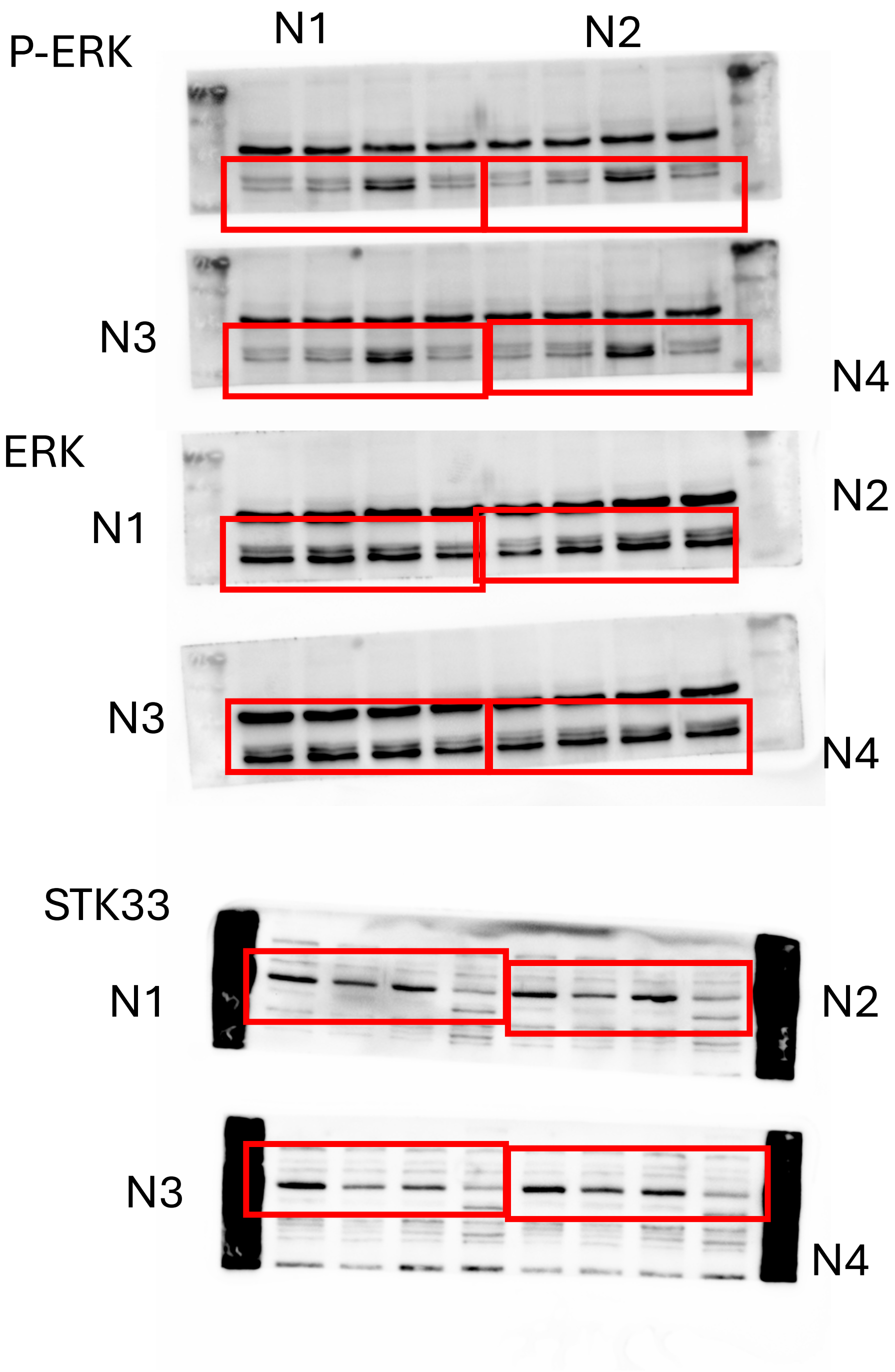
P-ERK
N1
N2
N3
N4
ERK
N1
N2
N3
N4
STK33
N1
N2
N3
N4

Fig 4B

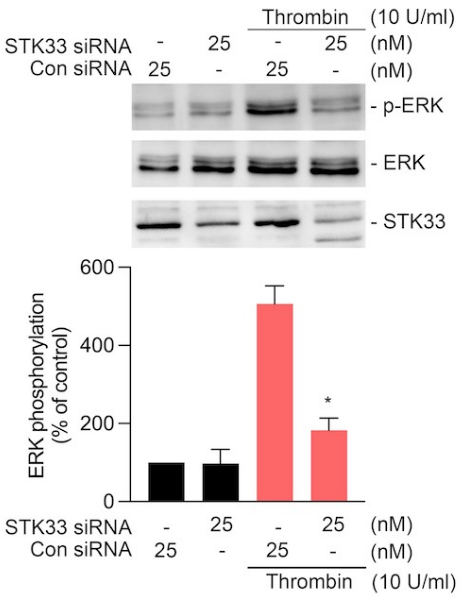

Thrombin (10 U/ml)
STK33 siRNA - 25 - 25 (nM)
Con siRNA 25 - 25 - (nM)
- p-ERK
- ERK
- STK33
ERK phosphorylation (% of control)
600
400
200
0
*
STK33 siRNA - 25 - 25 (nM)
Con siRNA 25 - 25 - (nM)
Thrombin (10 U/ml)

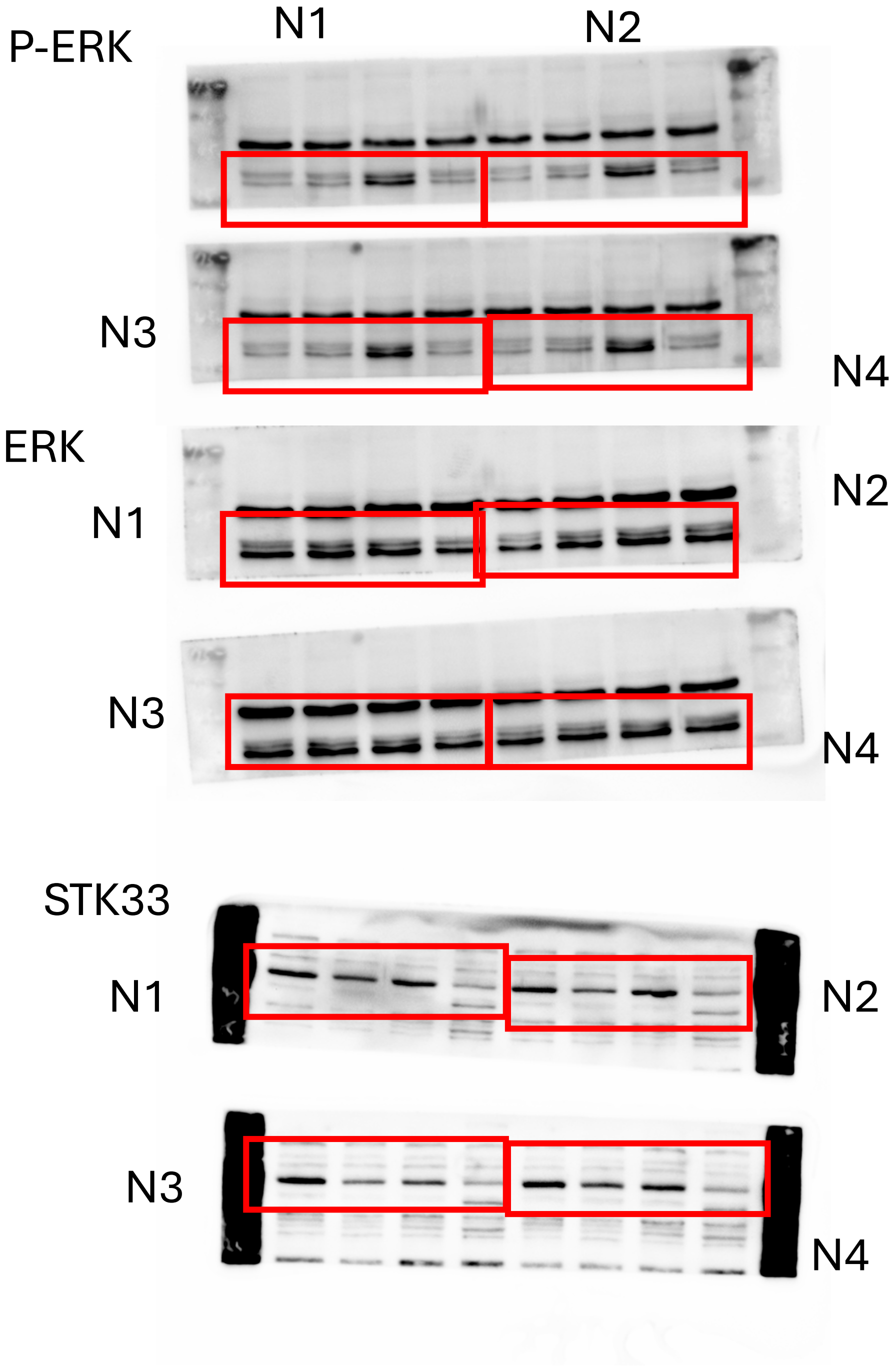

P-ERK
N1
N2
N3
N4
ERK
N1
N2
N3
N4
STK33
N1
N2
N3
N4

Fig 5E

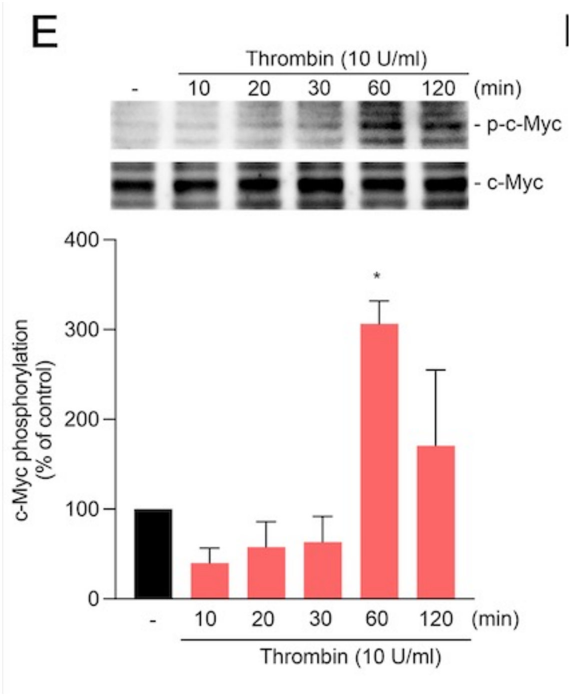
E
Thrombin (10 U/ml)
- 10 20 30 60 120 (min)
- p-c-Myc
- c-Myc
c-Myc phosphorylation (% of control)
400
300
200
100
0
*
- 10 20 30 60 120 (min)
Thrombin (10 U/ml)

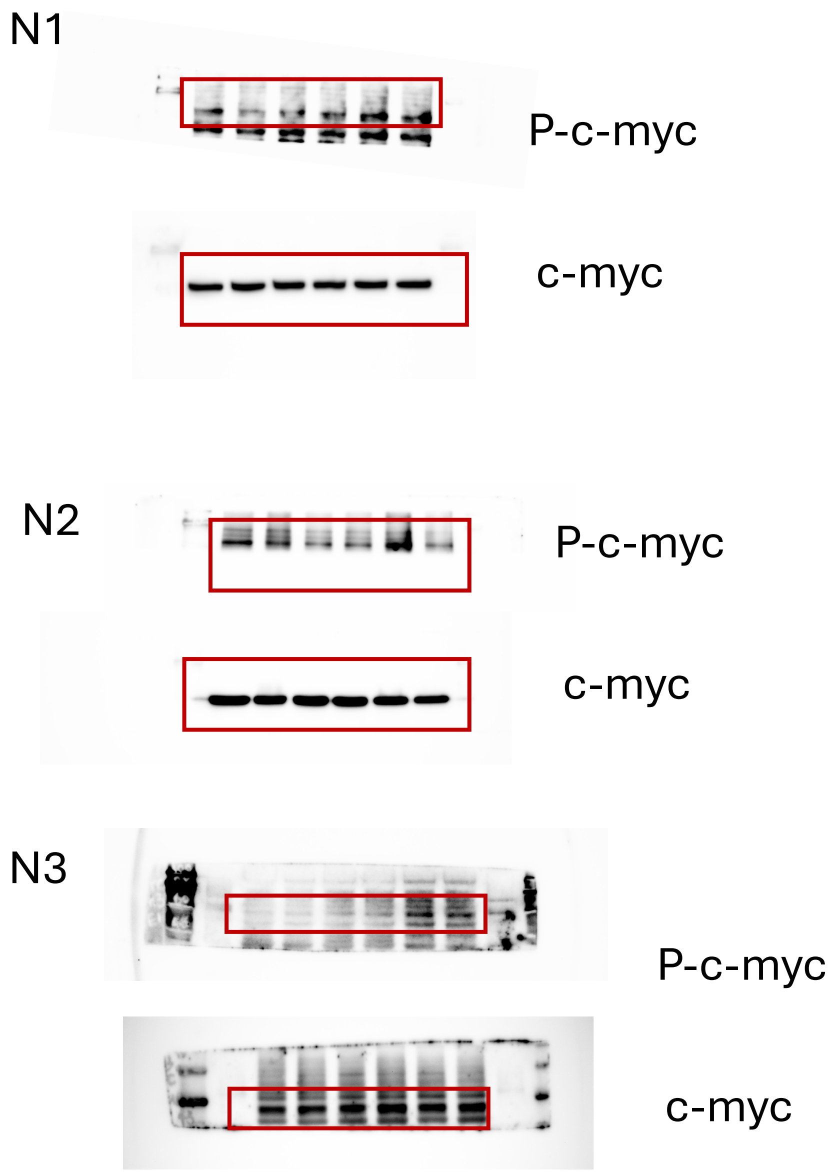
N1
P-c-myc
c-myc
N2
P-c-myc
c-myc
N3
P-c-myc
c-myc

Fig 5F

F

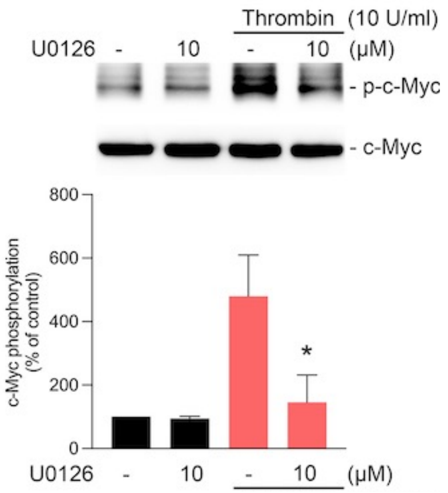

N1

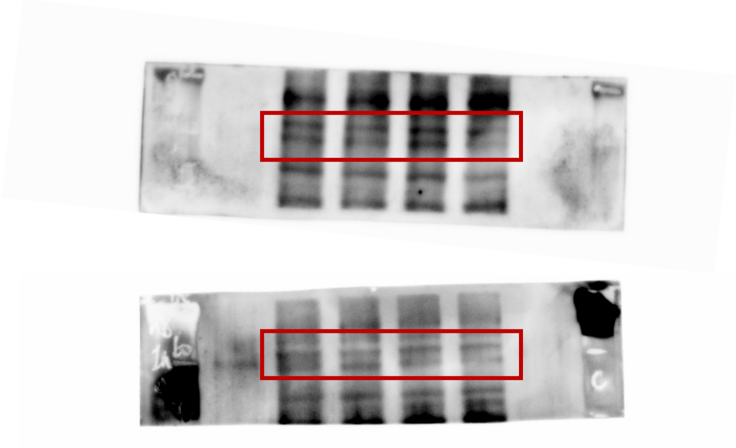

N2

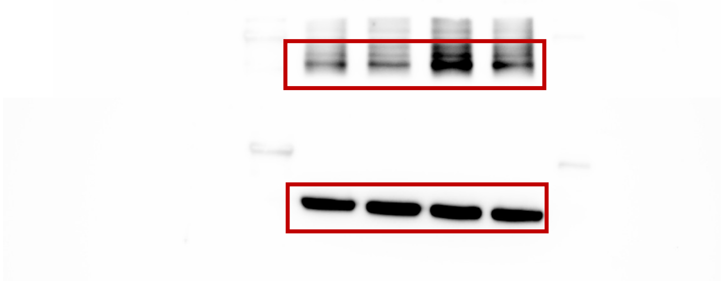

N3

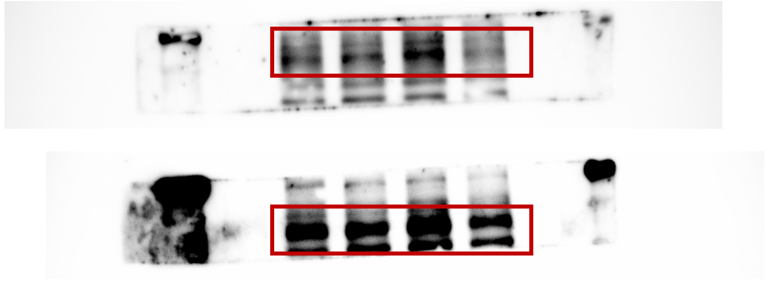

Fig 5G

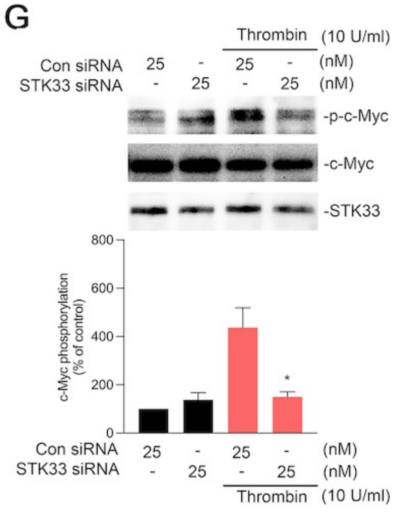
G
Thrombin (10 U/ml)
Con siRNA 25 - 25 - (nM)
STK33 siRNA - 25 - 25 (nM)
-p-c-Myc
-c-Myc
-STK33
c-Myc phosphorylation (% of control)
800
600
400
200
0
Con siRNA 25 - 25 - (nM)
STK33 siRNA - 25 - 25 (nM)
Thrombin (10 U/ml)

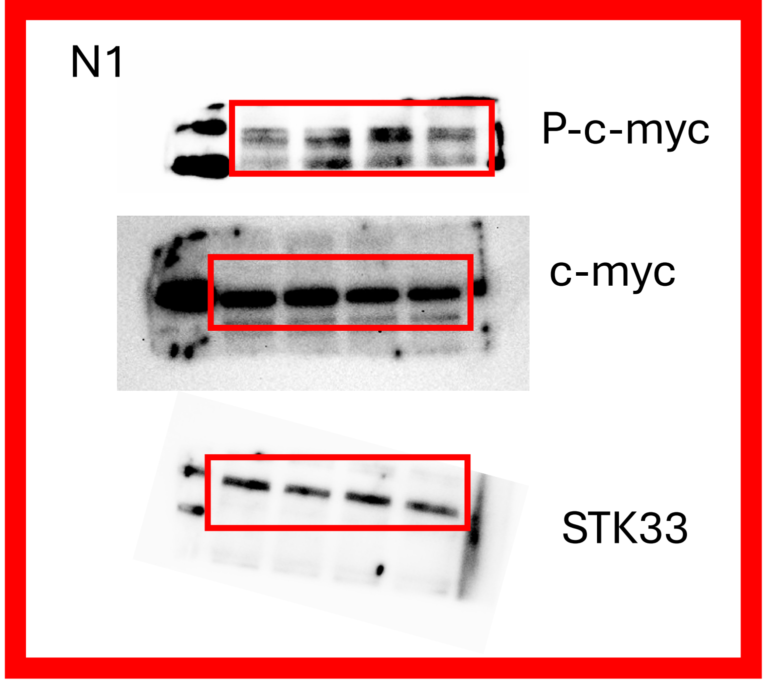
N1
P-c-myc
c-myc
STK33

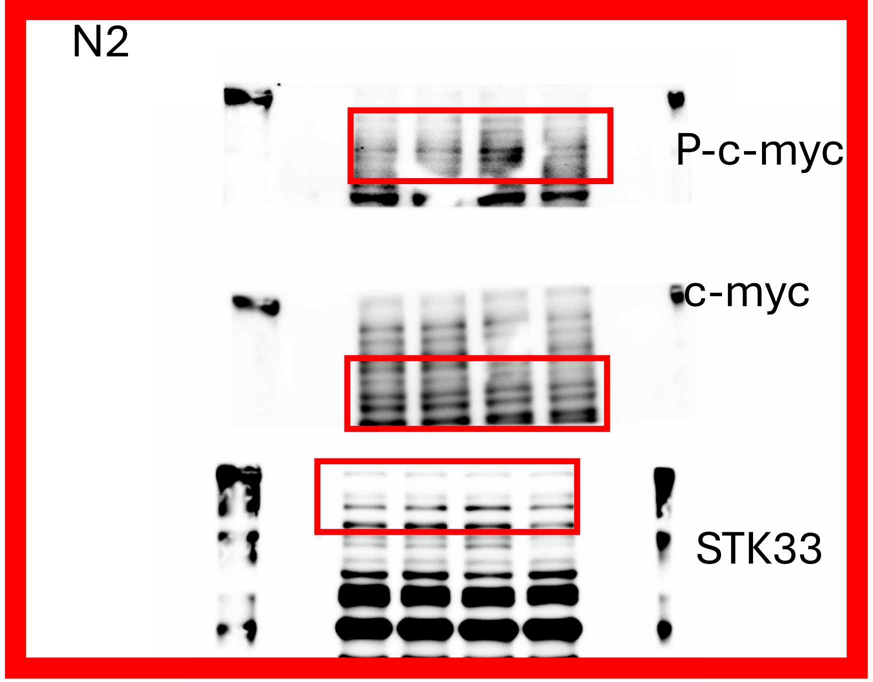
N2
P-c-myc
c-myc
STK33

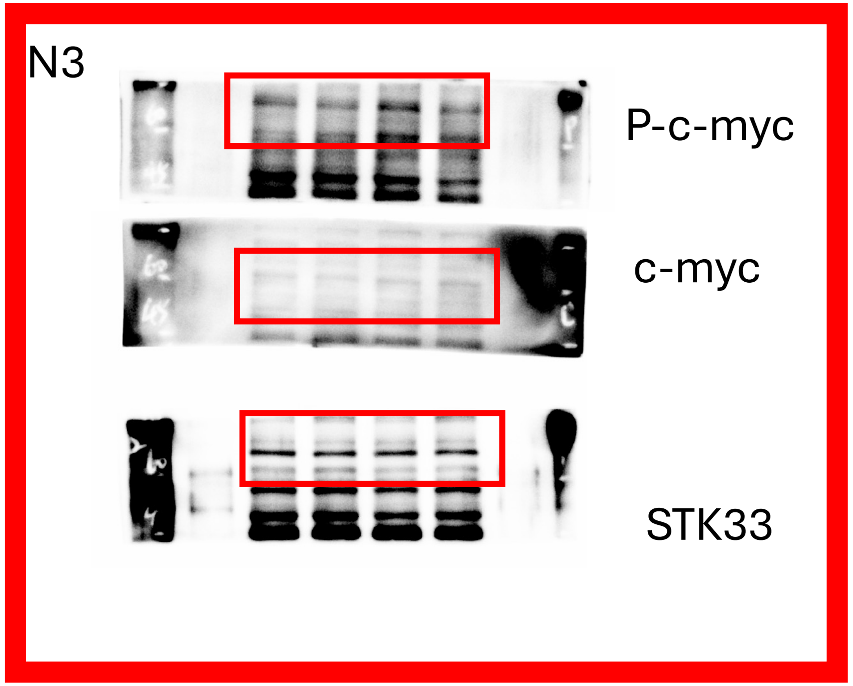
N3
P-c-myc
c-myc
STK33

Fig 6A

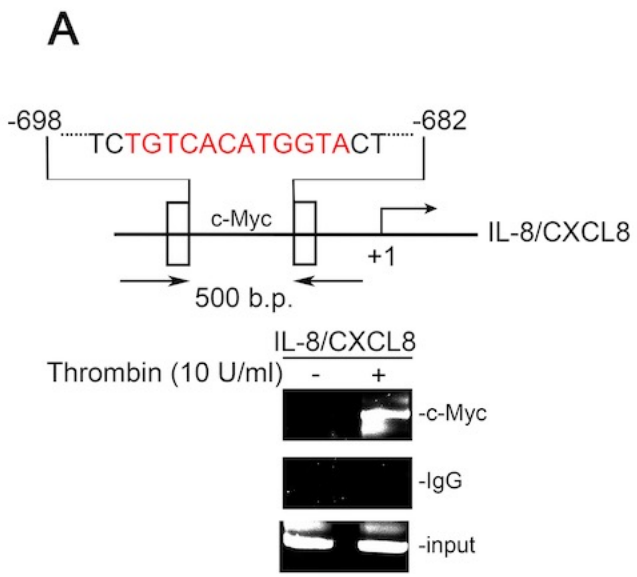
A
-698
TCTGTCACATGGTACT
-682
c-Myc
IL-8/CXCL8
+1
500 b.p.
IL-8/CXCL8
Thrombin (10 U/ml)
-
+
-c-Myc
-IgG
-input

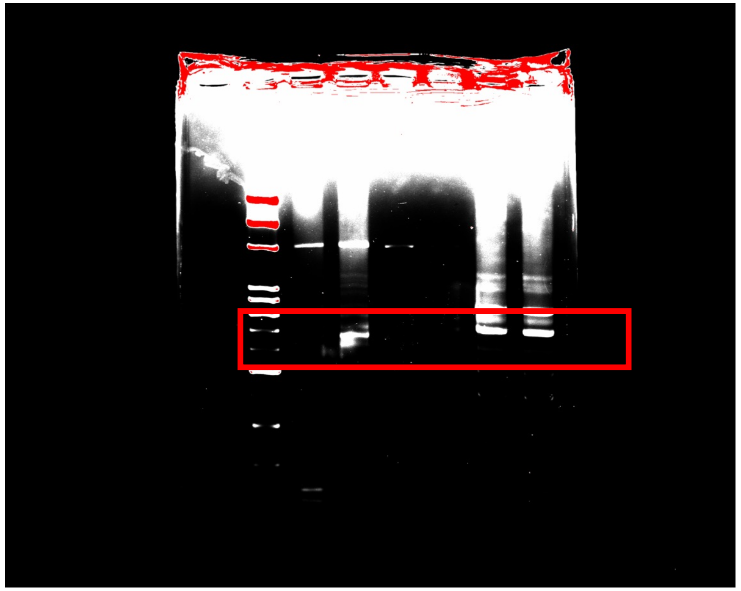

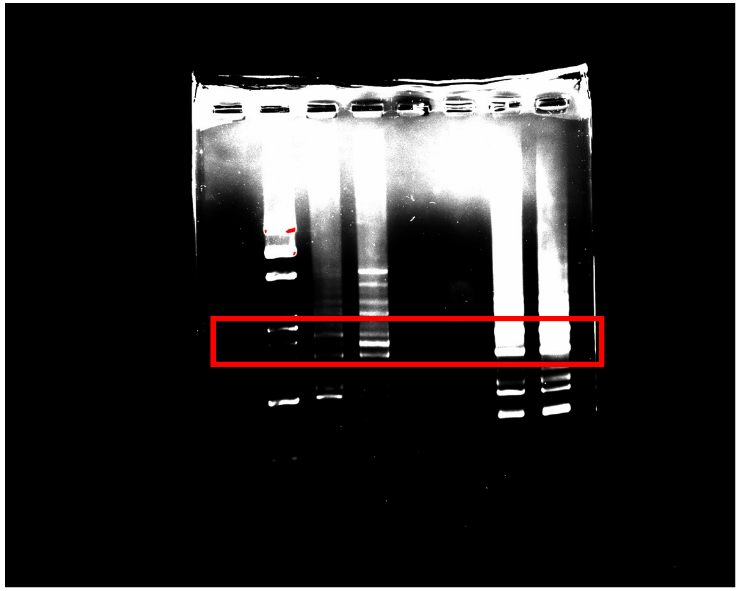

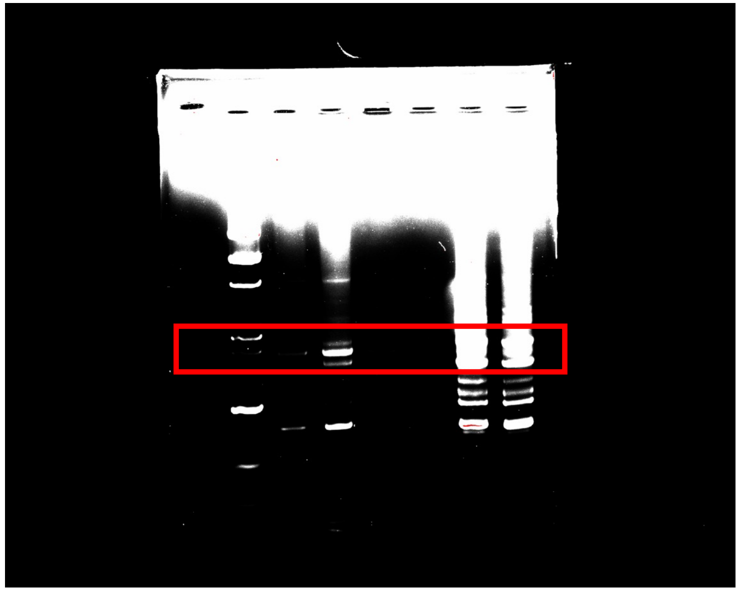

Fig 6D

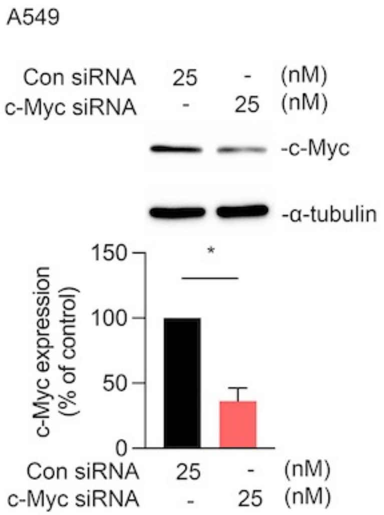

N1

c-myc

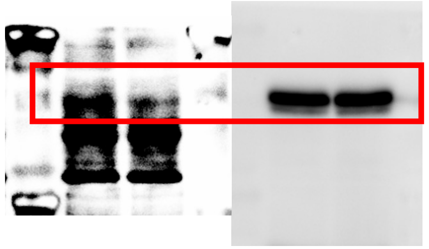

α-tubulin

N2

c-myc

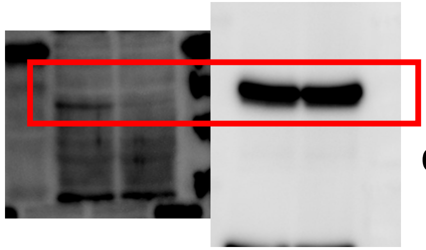

α-tubulin

N3

c-myc

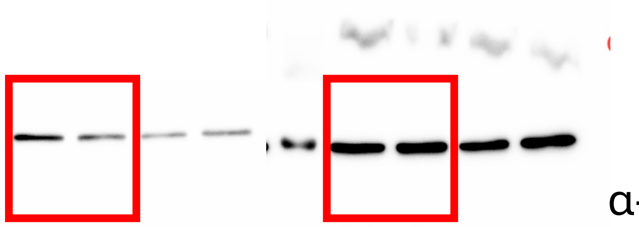

α-tubulin

Fig 6F

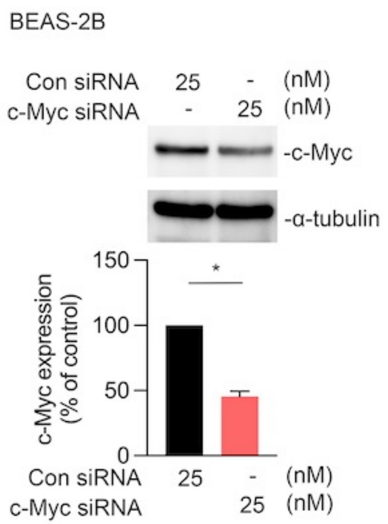
BEAS-2B
Con siRNA 25 - (nM)
c-Myc siRNA - 25 (nM)
-c-Myc
-α-tubulin
c-Myc expression (% of control)
150
100
50
0
*
Con siRNA 25 - (nM)
c-Myc siRNA - 25 (nM)

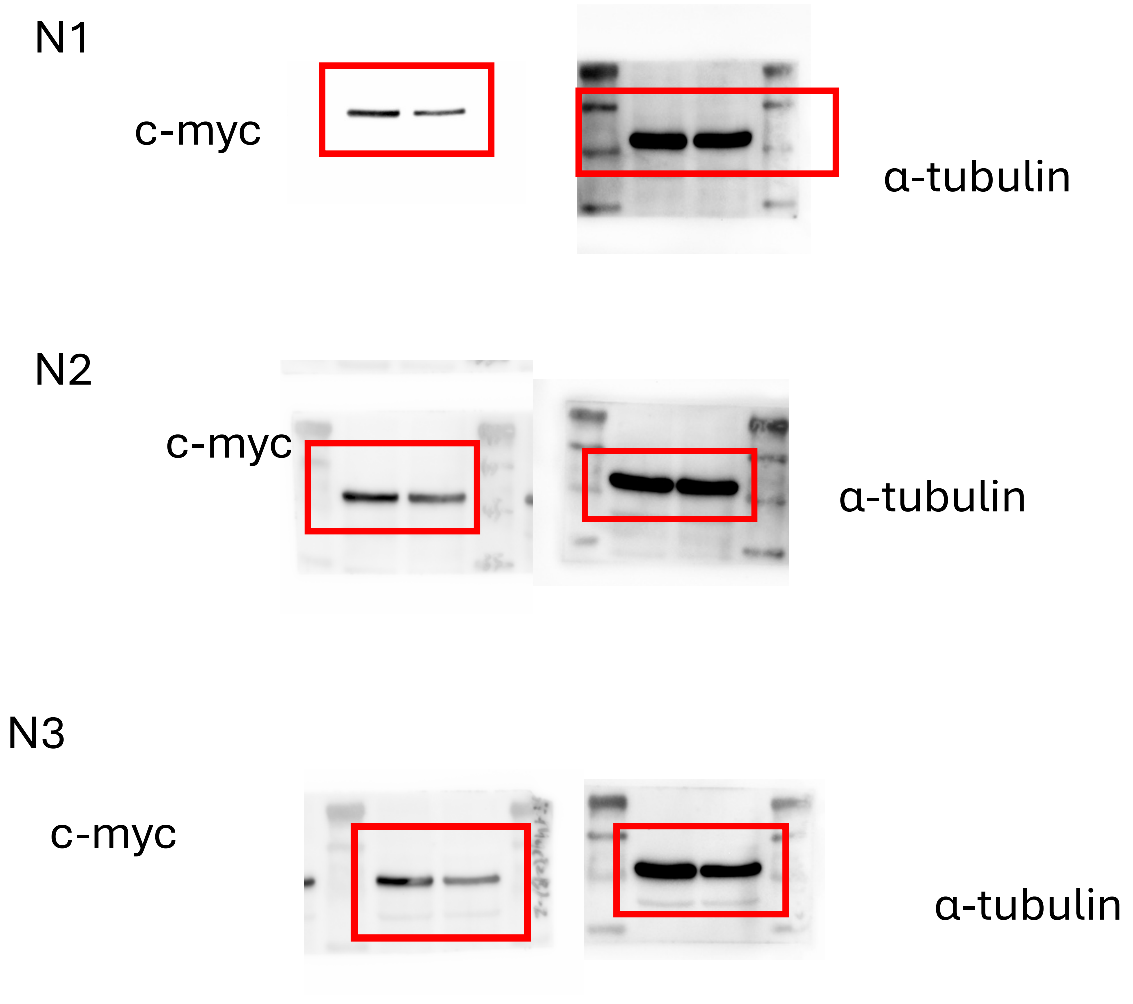
N1
c-myc
α-tubulin
N2
c-myc
α-tubulin
N3
c-myc
α-tubulin
